# Distribution and preservation of the components of the engulfment. What is beyond representative genomes?

**DOI:** 10.1371/journal.pone.0246651

**Published:** 2021-03-02

**Authors:** Lizeth Soto-Avila, Ricardo Ciria Merce, Walter Santos, Nori Castañeda, Rosa-María Gutierrez-Ríos

**Affiliations:** 1 Departamento de Microbiologia Molecular, Instituto de Biotecnologia, Universidad Nacional Autonoma de Mexico, Cuernavaca, Morelos, Mexico; 2 Centro de Investigacion en Dinamica Celular, Instituto de Investigacion en Ciencias Basicas y Aplicadas, Universidad Autonoma del Estado de Morelos (UAEM), Cuernavaca, Morelos, Mexico; University of Illinois at Urbana-Champaign, UNITED STATES

## Abstract

Engulfment requires the coordinated, targeted synthesis and degradation of peptidoglycan at the leading edge of the engulfing membrane to allow the mother cell to completely engulf the forespore. Proteins such as the DMP and Q:AH complexes in *Bacillus subtilis* are essential for engulfment, as are a set of accessory proteins including GerM and SpoIIB, among others. Experimental and bioinformatic studies of these proteins in bacteria distinct from *Bacillus subtilis* indicate that fundamental differences exist regarding the organization and mechanisms used to successfully perform engulfment. As a consequence, the distribution and prevalence of the proteins involved in engulfment and other proteins that participate in different sporulation stages have been studied using bioinformatic approaches. These works are based on the prediction of orthologs in the genomes of representative Firmicutes and have been helpful in tracing hypotheses about the origin and evolution of sporulation genes, some of which have been postulated as sporulation signatures. To date, an extensive study of these signatures outside of the representative Firmicutes is not available. Here, we asked whether phyletic profiles of proteins involved in engulfment can be used as signatures able to describe the sporulation phenotype. We tested this hypothesis in a set of 954 Firmicutes, finding preserved phyletic profiles defining signatures at the genus level. Finally, a phylogenetic reconstruction based on non-redundant phyletic profiles at the family level shows the non-monophyletic origin of these proteins due to gain/loss events along the phylum Firmicutes.

## Introduction

Bacteria have developed different strategies to survive. Several members of the phylum Firmicutes can form spores that are resistant to chemical and physical insults that remain dormant until favorable conditions allow the spore to germinate [1–4]. Genomic comparisons among Firmicutes, show a core set of genes that are almost conserved in all endospore formers [5–10]. These conserved genes are involved in sporulation as other less preserved genes control the complex process, which initiates with a signal transduction cascade induced by a variety of stress signal [11, 12]. The sporulation process in Firmicutes begins with a vegetative cell that produces an asymmetrical septum at one cell pole, in which a small compartment houses a forespore [13]. In *Bacillus subtilis* (*B*. *subtilis*), the forespore is then engulfed by the mother cell, which is a process that requires a thinning of the peptidoglycan layer of the septum to provide flexibility to the membrane and facilitate its migration around the forespore [14]. This function is carried out by genes that encode proteins to form the complex SpoIIDMP (DMP machinery) [15]. The expression of the proteins that form the SpoIIDMP complex is regulated by transcriptional regulators and sigma factors, which have been well studied in *B*. *subtilis* and *Clostridioides difficile* (*C*. *difficile*). These studies revealed differences in the roles and time of activity of the sigma factors and proteins of the complex during spore morphogenesis [16–19]. An example of these are the studies performed by Ribis et al., who propose that the expression of *spoIIP* encoding SpoIIP, under the control of σ^F^ in *C*. *difficile* and σ^E^ in *B subtilis*, may not be necessarily anchored to the forespore membrane throughout engulfment [20], which signifies a different organization and action mechanisms of the DMP machinery between both species and is a trait also suggested by other works [9, 15, 20, 21]. The engulfment in *B*. *subtilis* also requires at least nine other proteins that span the two opposing membranes that separate the mother cell and the forespore [22–24]. Eight proteins are encoded and organized in the operon *spoIIIA*, which is regulated by σ^E^ and expressed in the mother cell. SpoIIQ is the ninth protein produced in the forespore, and the expression of its gene (*spoIIQ*) is governed by σ^F^. It is also remarkable that the proteins encoded in the *spoIIIA* operon share a remote homology with different secretion systems observed in gram-negative bacteria [22–24]. It has been suggested that the Q:AH complex in *B*. *subtilis* functions as a novel secretion apparatus [25, 26].

Despite the substantial deviations from the *B*. *subtilis* model, particularly in the programs of gene expression in the forespore and the mother cell [27, 28], the components of the Q:AH complex seem to be highly conserved between both species [15, 21, 28–30]. Moreover, the comparative genomic approaches performed among representative genomes show that the genes that encode the Q:AH and DMP complexes, and other sporulation genes present several evolutionary steps, with gene gains/losses, being the key events for the specialization of this developmental program and the adjustment of bacteria to particular ecosystems [5–8, 29]. In the case of the Q:AH and DMP complexes, experimental evidence indicates their interaction with other gene products, such as the GerM protein implicated in germination, and that is known to interact or affect the Q:AH complex in *B*. *subtilis* [31]. SpoIIB is also necessary in *B*. *subtilis* for the efficient degradation of septal peptidoglycan [32, 33], which is not the same in spore formers such as clostridial and members of the family *Alicyclobacillaceae* lacking SpoIIB and post‐septation proteins [29].

Notwithstanding the differences found in the mechanisms and distribution of the genes involved in engulfment, some of them have been classified as sporulation signatures, as derived from the inspection of genomes of representative Firmicutes [5, 7, 10, 29]. The differences in the distribution found in some representatives motivated us to investigate the distribution and prevalence of the Q:AH and DMP complexes and the SpoIIB and GerM proteins, beyond representative Firmicutes, to corroborate if these proteins represent a machinery specific to endospore formers in this phylum. The establishment of the distribution and prevalence of genes among genomes is based on a comparative genomic analysis that predicts the presence of orthologs, such as those involved in sporulation [17, 29, 34, 35]. A previous work of ours [36] and another by Davidson et al. [37] based on the scanning of Hidden Markov Models profiles (HMM-profiles) in bacterial proteomes and the preservation of key genes in the genomic context have proven to be a powerful method to detect nearby and distant probable orthologs (POs). We tested this approach on the proteins of the phosphorelay initiating sporulation in Firmicutes [36]. The approach was a determinant to identify the transferases Spo0B and Spo0F in some species of Clostridia, which was considered for a long time to be a two-component system composed only of an orphan histidine kinase and the response regulator Spo0A [1, 18, 38].

In the present work, we used our aforementioned method to construct protein architectures based on HMM-profiles, which describe the proteins that participate in the engulfment such as accessory proteins that help to assemble the Q:AH and DMP complexes in *B*. *subtilis*. With the constructed protein architectures, we searched for the POs in the proteomes of a set of 4,852 fully sequenced genomes, and found that the POs are confined into Firmicutes, and as previously described, are extensively distributed into endospore formers. The sporulation and nonsporulation phenotypes for this investigation were manually curated from the literature, which revealed that of the 954 Firmicutes, 26% of the phenotypes were inferred from other species that belong to the same genus and 8% of the species have an unknown phenotype.

As mentioned before, the prevalence of the proteins of the engulfasome among endospore formers, has caused them to be considered as sporulation signatures, and some of them prevailing up to 100% in some of the representative Firmicutes genomes [7, 8, 10, 29]. Our results show that the representatives chosen to determine the prevalence of the orthologs that control sporulation in several cases do not reflect the preservation of the ortholog even between strains. However, as has been stated, the preceding discussion does not prevent determining a minimum set of genes that defines each stage of sporulation. Moreover, the results obtained in this work for the first time allow offering specific signatures to each genus based on the observed phyletic profiles. Additionally, the prediction of POs through our method and the construction of phyletic profiles are demonstrated to be a valuable tools that allowed the recognition of incorrect annotations. Finally, a selection of species based on nonredundant phyletic profiles used to reconstruct a phylogenetic tree results in a consistent clustering of species across genera and families, which enables us to estimate gain and loss events, which were necessary to adapt the Firmicutes genomes to the ever-changing environments.

## Methods

### Datasets

The genomic data for 4,852 completely sequenced bacterial genomes were retrieved from the KEGG Database (https://www.kegg.jp/) in April 2019 [39]. The database at this time stored the genomes of 954 Firmicutes, for which a sporulation phenotype was assigned as described later in the Methods section. From the KEGG-GENOME database, we extracted the taxonomy and the phylogenetic lineages taken by KEGG from the NCBI RefSeq repository (https://www.ncbi.nlm.nih.gov/taxonomy). We also extracted the amino acid sequences of each protein, and the KEGG gene identifier. From the KEGG orthologs (KOs) database [40], we extracted the groups of KOs.

### Construction of protein architectures

**Fig 1:**
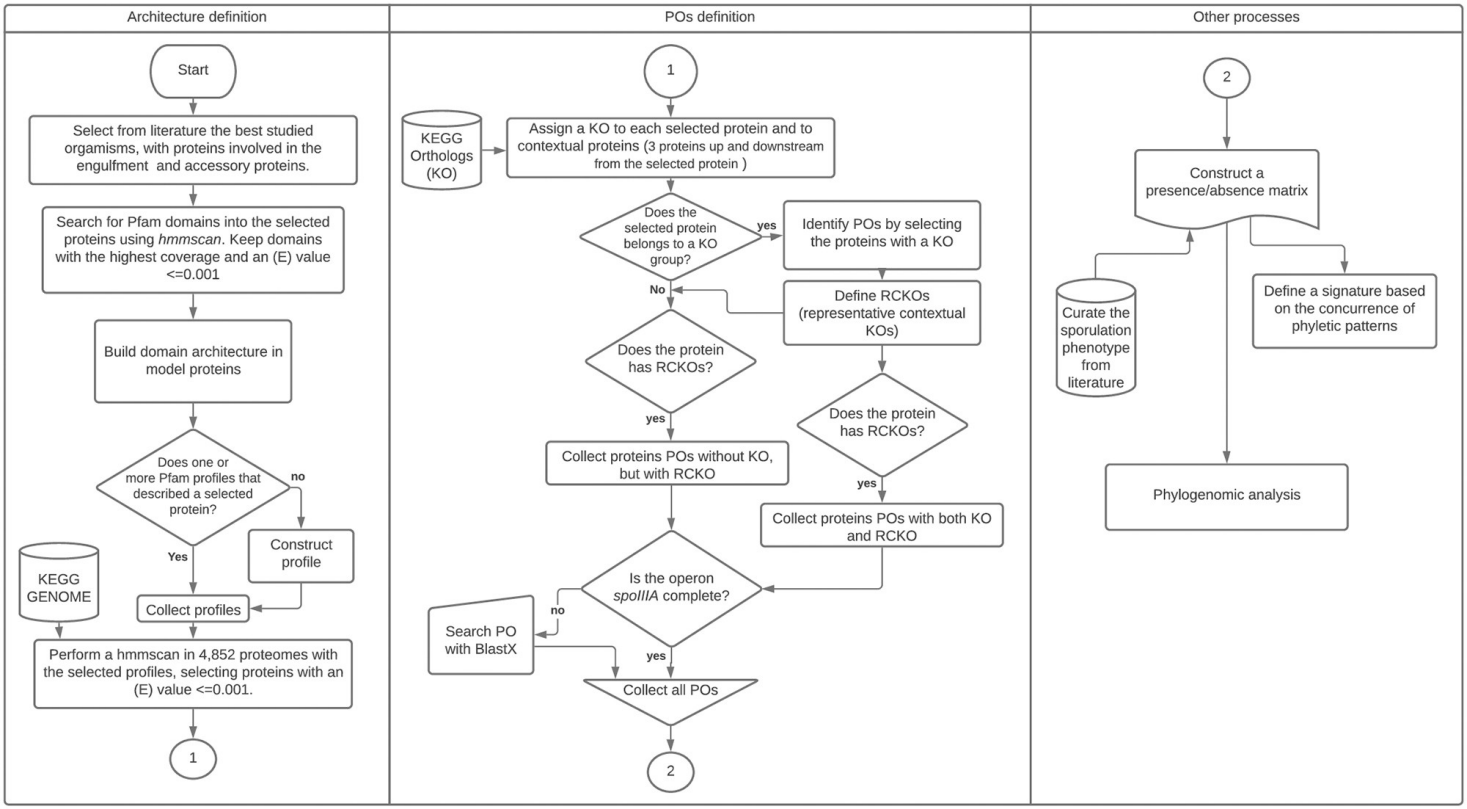
Identification of the PO. Overview of the method proposed to predict the PO, the construction of phyletic profiles and the phyletic profile signatures.

The identification of POs is based on our own methodology [36] in which the first step, as shown in Fig 1, is to define the protein models, by consulting the pertinent literature to identify the proteins experimentally proven to be involved in the engulfment. We found that experimental evidence is available for *C*. *difficile* and *B*. *subtilis*. The selected proteins that serve as models, were those of the complexes Q:AH and DMP, the proteins SpoIIB, which were suggested to regulate the septal thinning during engulfment in *B*. *subtilis* [33], and GerM which is required to assemble the basal platform of the Q:AH complex also in *B*. *subtilis* [31].

The KOs of the model proteins were retrieved, and all the sequences related to the KO were employed to create a database in which we performed a *hhmscan* with HMMER v3.1b1 [41] using the profiles of the protein families stored in the curated PfamA database [42]. We preserved the sequences with the domains that have an expectation (E) value < = 0.001 since Pfam considers 0.001 to be a significant cutoff. This cutoff is actually used to reconstruct the clans and protein families in their update published in 2019 [42]. Then, we identified the domain that covers the longer length of each protein, and we found that some protein sequences, have two or more overlapping domains. When a protein presents two or more overlapped domains, we selected the domain that encompassed the longest length and a significant (E) value. An example is SpoIIQ, which has two domains: the RnfC barrel sandwich hybrid domain and the Peptidase family M23 [43, 44] that encompasses the RnfC domain. Therefore, the Peptidase family M23 was selected as the representative domain, which was previously used for ortholog identification [28].

**Fig 2:**
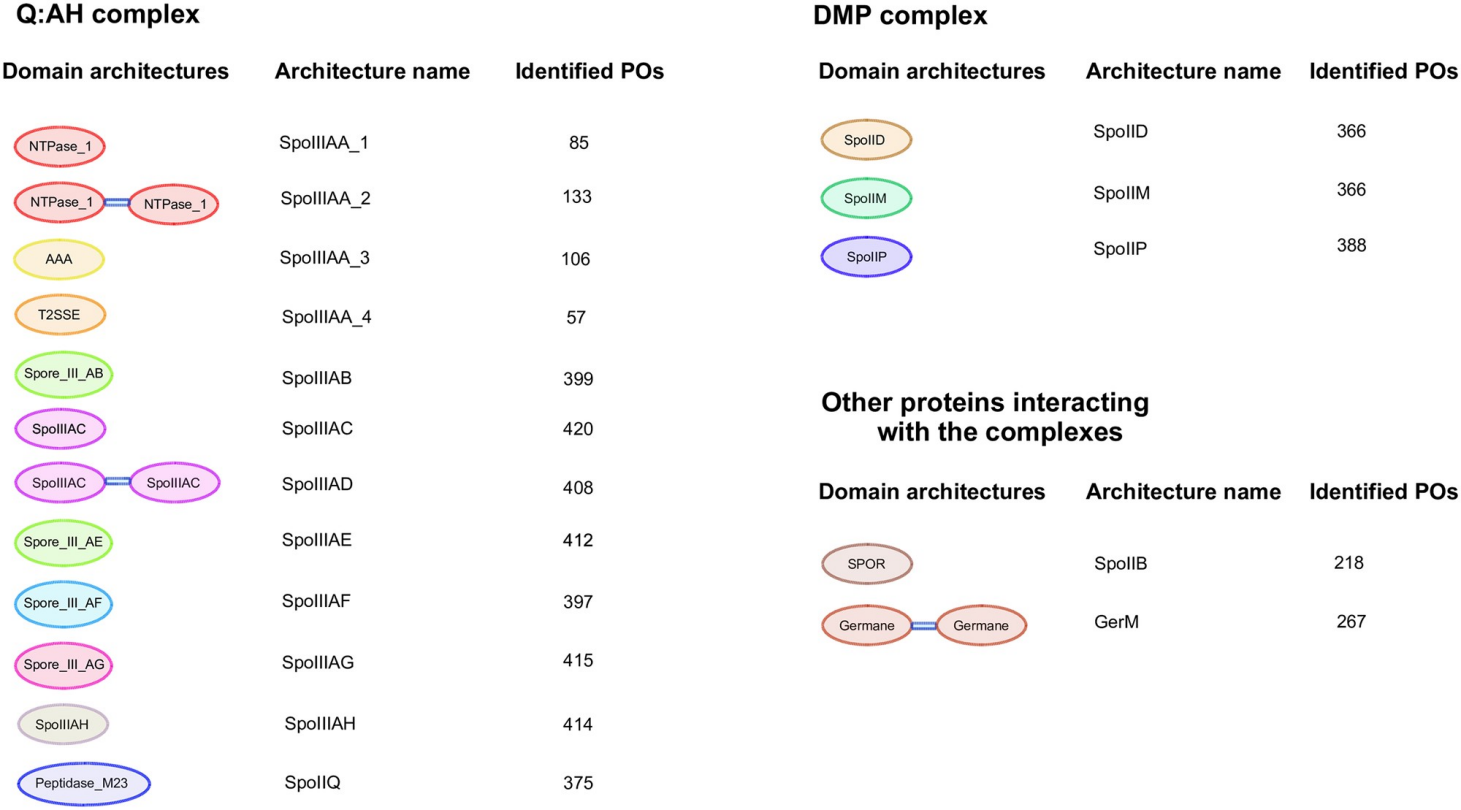
Architectures of the Q: AH and DMP complexes and accessory proteins. Pfam architectures that were used as models to scan for the PO in bacterial proteomes and its frequency of appearance in Firmicutes.

As some of the Pfam domains hold structural or sequence motifs, we also used this information to construct the protein architectures, for example, the protein SpoIIIAA of the Q:AH complex, which is a typical ATPase that preserves the Walker A and Walker B motifs, and the His and Asp boxes in *B*. *subtilis* and *C*. *difficile* [25, 28]. We found that this protein could be represented by 229 Pfam profiles grouped in the P-loop_NTPase clan [42]. From this, according to the KO, the most extensive length, and an (E) values < = 0.001, we selected the profiles PF00004 (AAA), PF00437 (T2SSE), and PF03266 (NTPase_1), which were organized in four different architectures as shown in Fig 2.

The inspection retrieved 14 Pfam profiles that can identify all the proposed proteins (Fig 2). In the case of the SpoIIIAG protein, we built a specific profile since the Pfam Spore_III_AF (PF09581), equally identified the sequences related to the SpoIIIAF and SpoIIIAG proteins. The profile was built with 27 nonredundant sequences selected by running the Cd-hit program [45], with a cutoff threshold of 80%. The seed group consisted of 73 sequences that have K06396. The 27 selected proteins were first aligned using MUSCLE [46] and converted to a Stockholm format using the online converter program reported in (http://sonnhammer.sbc.su.se/Stockholm.html). We then used *hmmbuild*, which is part of the HMMER v3.1b1 suite [41], to construct a profile for SpoIIIAG using hidden Markov models and default parameters. The SpoIIIAG profile (see File 1 via https://doi.org/10.6084/m9.figshare.13338677.v1) was then used to perform a *hmmscan* search.

### Identification of POs using the genomic context

The identification of the homologs that correspond to a KO [40] is the first step to discern the paralogues. As expected, this is not the best criterion to identify POs since not all the proteins have an assigned KO. Considering this observation, we inspected the genomic context of the model proteins (see S1 Table in S1 File), as proposed in our previous work [36]. We limited the context to three genes upstream and downstream from the reference protein. To simplify the context inspection, the neighbor genes were also classified by their KOs. We selected as representative contextual KOs (RCKOs) the KOs that appear at least eight times (an arbitrary cutoff), and the full list of contextual KOs and their frequencies can be consulted in S2 Table in S1 File. The next step was to search for the RCKOs upstream and downstream from a protein with a predicted architecture. As expected, some of the homologs that have an architecture and others found in the RCKOs do not possess a predicted KO as shown in S3 Table in S1 File. For the predicted homologs in which the KO was missing the RCKO was used to define a PO. For the predicted homologs where at least one contextual KOs was not defined, the prediction was dismissed. In S2 Table in S1 File, we show each KO’s frequency in the genomic context.

The final rules used to consider a protein as a PO were as follows. 1. The protein should have a defined architecture that includes the most extended domain, and when known, the domain or domains should include the protein sequence motifs. 2. The protein should have at least one RCKO. 3. When a KO was assigned to the query, we used it, but this condition was not required. The selected RCKOs and KOs of the target genes are shown in S4 Table in S1 File. The results of the predictions that use the architectures and the rules mentioned above were represented in a presence/absence matrix of phyletic patterns in which rows correspond to species, and columns correspond to binary characters; “0” stands for absence, and “1” stands for presence (S5 Table in S1 File).

### Sporulation phenotype compendia

In this work, we curated the sporulation phenotype of the 629 Firmicutes. The other 325 phenotypes from the 954 Firmicutes were recently published by our group [36]. To this end, we used two methods to acquire information. First, we searched the databases that offer curated phenotypes such as the Interactive Atlas for Exploring Bacterial Genomes (BacMap), the Genomes OnLine database (GOLD) and the Pathosystems Resource Integration Center (PATRIC). Second, we inspected the literature for the missing organisms in these databases [47–49]. Additionally, we searched for recent information to corroborate the phenotypes offered by these databases and found that most of the entries were recently updated. The curated phenotypes were added to the presence/absence matrix shown in S5 Table in S1 File. We also included in the matrix the links to the manuscripts or databases from which the phenotypes were curated.

### Annotation of missing *spoIIIA* genes

We conducted a BlastX sequence search [50] in the upstream regions of the missing proteins of the *spoIIIA* operon into the genomes of the curated endospore former Firmicutes. We considered it to be a significant hit when the searched protein has as the best match the protein of the query organism or a nearly related species or a hit on multispecies, which included the searched species. We then scanned the translated sequences with the Pfam profiles stored in the PfamA database. In this case, we used an (E) value < = 0.01 since we wanted to recover the hits not found in the first *hhmscan* round. Despite the cutoff, the profiles always gave significant matches when found. The SpoIIIAA sequences that matched were aligned and inspected because we wanted to preserve the proteins that have the Walker A and B motifs and the His and the Asp boxes.

### Methods for the identification of phyletic patterns signatures

To estimate the preservation of the phyletic patterns within each genus, we calculated a measure of aggregation or segregation, as evaluated by a Checkerboard score (C-score), which was originally used to measure the association between species pairs and that was adapted to test the presences (1) and absence (0) matrix of a protein (column) in a species (row). Thus, for any particular species/protein pair, the C-score is a numerical index that ranges from a maximally aggregated (a minimum of 0) to a maximally segregated with no shared proteins in the profile. [51]. We used the module cooc_null_model of the EcoSimR package, developed in R [51]. The EcoSimR package in our case conducted a protein cooccurrence analysis using null models, with parameters “sim9” and “c_score” that have satisfactory performance with most types of random matrices (= low Type I errors). As stated before, in the input data were the presence/absence matrix of each phyletic profile per genus with a number of species > = 2. We considered a positive result to be all the genus matrices that possess an arbitrary C-score < = 0.2.

### Species tree construction

As a first step, all orthologous proteins of 168 organisms that have a distinctive phyletic profile into its family were identified with the bidirectional-best hit (BDBH) method when comparing proteomes using BLASTp [50], with a cut-off value of (E) value <1e-04. To consider that two proteins were orthologous, in addition to the BDBH criterion, the coverage of the region of similarity identified by BLASTp [50] should cover at least 50% of the length of the smallest protein of the pair of proteins compared. Once the set of orthologous proteins were identified, for the construction of the mega-alignment, these proteins found in at least 90% of the set of study organisms were chosen (see File 3 via https://doi.org/10.6084/m9.figshare.13338686.v1). These sequences were aligned using the MUSCLE program [46]. For all the alignments to have the same number of elements, the missing protein sequences in the alignments were replaced by a chain of dashes of the same length as that of the substituted proteins. Subsequently, the columns of the alignments that did not have at least 90% of the informative positions were eliminated. Finally, all the alignments were concatenated to form the mega-alignment that was used for the phylogenetic analysis. To reconstruct the tree, we used IQ-TREE multicore version 1.6.11 for Linux 64-bits, (maximum likelihood) with the aLRT correction (a standard likelihood ratio test approximation) with 1,000 replicates to find the best bootstrap distance model [52]. The tree that grouped 168 species was visualized and rooted with the iTol tool [53].

### Gain and loss analysis

We used the Gain Loss Mapping Engine (GLOOME) to estimate the number of gain and loss events that have occurred across the Firmicutes chosen to construct the species tree over the course of the evolution of the proteins involved in engulfment [54]. The gain-loss analysis implemented by GLOOME integrates the presence-absence data for each gene of interest across the phylogenetic profile to estimate the posterior expectation of gain and loss across all branches. These events are then summed to calculate the total number of gene gain and loss events that have occurred for each family across the phylogenetic tree. We performed this analysis using the mixture model with a variable gain/loss ratio and a gamma rate distribution. The interquartile distance distribution of a branch length was calculated and used as a parameter to organize the gain and loss events from the root to the tips. This means that the first quartile (q1 25%) groups the shortest distances from the root, and q4 (75%) groups the longest distances.

## Results

### Identification of the probable orthologs involved in the engulfment

At present, the number of organisms that can be currently analyzed has grown considerably, which makes it possible to exhaustively compare the genome composition of a vast number of organisms. In this work, we performed a comparative genomic approach to investigate the phylogenetic extent of the proteins involved in engulfment and other proteins necessary to assemble two of the complexes that form the engulfasome beyond the representative Firmicutes. Currently, Firmicutes are divided into seven classes, namely, Bacilli, Clostridia, Negativicutes, Erysipelotrichia, Limnochordia, Thermolitobacteria, and Tissierellia (source: NCBI taxonomy), but since we are working with fully sequenced genomes, the analysis excluded Thermolitobacteria.

To perform the analysis, we employed a methodology of our own that can discover distant POs based on the construction of protein architectures using Pfam profiles that describe the proteins of the Q:AH and DMP complexes that form a structure recently named engulfasome [9] and the accessory proteins SpoIIB, which is suggested to regulate the septal thinning during engulfment in *B*. *subtilis* [33], and GerM that probably localizes SpoIIQ in the Q:AH complex also in *B*. *subtilis* [55]. The procedure yielded 17 architectures sufficient to identify both complexes and the two accessory proteins. The profiles described in the Pfam database identified the homologs of sixteen of the seventeen proteins, except for the SpoIIIAG part of the Q:AH complex. SpoIIIAG displays remote homology to the “Ring Building Motif (RBM)” also present in the homologs of SpoIIIAF and SpoIIIAH [24]. To resolve this ambiguity, we constructed a profile for SpoIIIAG and found 415 hits, distinct from those proteins with a predicted SpoIIIAF and SpoIIIAH.

Once an architecture was assigned, the genomic context was inspected, assuming that the genes that are most similar at the sequence level should retain the ancestral gene-neighborhood after duplication [56]. We also assumed that some contextual information will be consistent with that found in the genomes of *B*. *subtilis* and *C*. *difficile* (see S1 Table in S1 File) organisms whose engulfment components are also well studied. We also defined the presence of other proteins frequently represented in the genomic context of homologs with a predicted KO (S2 Table in S1 File), which is a decision that may leave out some true positives, in which the context is poorly defined. Therefore, the selection of the final set of POs need both a) the prediction of a valid architecture and b) the presence of a valid genomic context. The application of these rules also allowed us to identify 297 POs with a KO not assigned (see S3 Table in S1 File). Homologs without a KO were found in the entire set of POs, except for the group of homologs defined by the architectures of SpoIID and SpoIIP.

At the end of the process shown in Fig 1, we proposed seventeen architectures that can be consulted in Fig 2, which also shows the number of predicted POs in the Firmicutes proteomes. From these, 15 architectures were constructed with a unique domain; however, proteins such as SpoIIIAA are represented by more than one Pfam. We identified four architectures that define SpoIIIAA; the SpoIIIAA_1 architecture was constructed with the NTP_1 domain (PF03266), the SpoIIIAA_2 architecture uses the NTP_1 domain in the tandem copy (PF03266), the SpoIIIAA_3 architecture is represented by an AAA profile (PF00004), and the SpoIIIAA_4 architecture uses the T2SSE (PF00437) Pfam. In the case of SpoIIIAA_2, the tandem copies of NTP were necessary to cover the Walker A and B motifs and the His and Asp boxes [25]; the detailed results of the Pfam organization for each species are shown in the S6 Table in S1 File. Other proteins that need tandem copies for PO recognition are GerM, which was previously reported to have two copies called GerMN1 and GerMN2 [55], described by the Pfam profile (PF10646) called Germane. Another protein that need a tandem copy for a full detection was SpoIIIAD that has SpoIIIAC (PF06686) in tandem copies.

As we demand that the domains that define SpoIIIAA have the Walker motifs, and the His and Asp boxes, we aligned the *hhmscan* hits, and found 43 proteins in which the profile just covered the Walker A or Walker B motifs. Nonetheless, the inspection of the alignment showed that the rest of the motifs were present in the protein sequences, except for two species of Clostridia, specifically, *Heliobacterium modesticaldum* and *Clostridium botulinum* H04402 065, which have two contiguous small proteins that hold the expected motif.

### Distribution of the probable orthologs beyond the representative Firmicutes

We searched for the PO involved in engulfment by inspecting, the 4,852 bacterial proteomes, which were found only in the proteomes of 954 Firmicutes. The results were then organized in a presence/absence matrix of phyletic profiles, which were associated with a curated sporulation phenotype (S5 Table in S1 File). The observed phenotype frequencies grouped by the phylogenetic class are presented in Table 1. The table is also organized by considering the types of evidence that describe the phenotype. As a result, two groups show the number of species with phenotypes and present rigorous experimental evidence which was as sporulating or nonsporulating. Two other groups show the species for which the projects described a sequenced genome, whose sporulating or nonsporulating phenotype was inferred from typical morphologies that describe the family or genus, as in the case of the *Streptococcaceae* family described as a group of non-endospore formers [57]. Finally, we found a fifth group that included organisms with unknown phenotypes, and this group has members in which in the family or genus of both sporulating and nonsporulating species have been observed. Both nonsporulating types include “asporogenic” organisms, which are defined as bacteria with an impaired sporulation process but contain the majority of sporulation genes, and “nonspore-forming” that include organisms with an absence of sporulation-specific genes [5].

**Table 1:** Curated sporulation phenotypes among Firmicutes.

Class	Sporulating	Sporulating- inferred	Nonsporulating	Nonsporulating-inferred	Unknown
Bacilli	187	28	245	214	53
Clostridia	114	4	56	2	19
Erysipelotrichia	0	0	6	0	3
Negativicutes	2	0	9	3	3
Limnochordia	1	0	0	0	0
Tissierellia	1	0	2	0	2
Total	305	32	318	219	80

The table shows the distribution of the curated phenotypes of 954 manually curated species of Firmicutes. Column 1 shows the classes that group the curated species. The frequency by class of the sporulating and nonsporulating species are shown in columns 2 and 4, which describe the species that were tested in wet laboratory conditions to define the phenotype. Columns 3 to 5 show the total number of sporulating and nonsporulating phenotypes by class as inferred from other species, and column 6 presents the frequencies of the class of species whose phenotype is not yet defined. At the end of each column, we preset the total number of species that have the curated phenotype.

**Fig 3:**
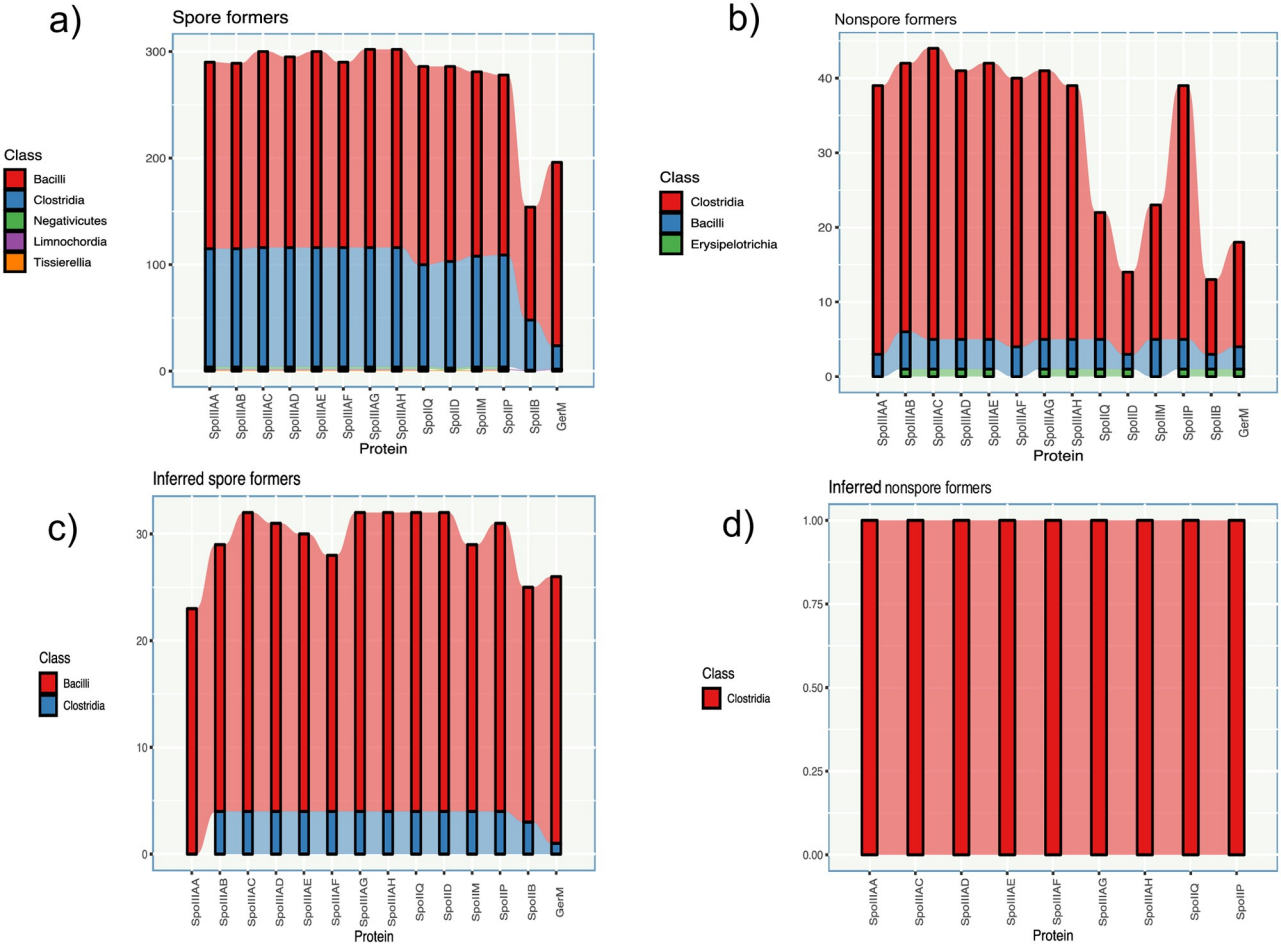
Distribution of the POs among phenotypes. a) Distribution of the POs among spore formers. b) Distribution of the POs among nonspore formers. c) Distribution of the POs among inferred spore formers. d) Distribution of the POs among inferred nonspore formers. Each figure shows the number of POs identified for each protein grouped by phenotype and by phylogenetic class.

Both curated phenotypes and POs were used to analyze the distribution of the studied proteins. The results presented in Fig 3a show that the endospore formers have the highest proportion of preserved POs (median = 290 POs from 305 species), but this proportion decreases in non-endospore formers (median = 29.5 POs from 318 species), as seen in Fig 3b, which is an observation that is consistent with the findings reported by Galperin [6, 29], Abecasis [7] and recently, Ramos-Silva [8]. Previous studies and ours also show that even in proteins whose genes are structured in operons, as is the case of *spoIIIA*, the ortholog proteins are not 100% conserved (media = 297.5, n = 305). The POs of SpoIIQ (286 from 305) and the DMP complex (median = 281, n = 305) are also preserved in corroborated endospore formers, but as seen, the orthologs of the DMP complex tend to be absent in almost 24 species. The POs of SpoIIQ, SpoIID, and SpoIIM were significantly less identified (median = 22.5, n = 318) than those of the POs of *spoIIIA*.

We also separately analyzed the distribution of the POs in 251 species without an experimentally confirmed phenotype. These species that have an inferred sporulating phenotype (n = 32) (Fig 3c) have more than 75% of the calculated POs, except for *Sporosarcina* sp. P37, *Sporosarcina* sp. P33 and *Lysinibacillus fusiformis* RB 21, which have a PO prediction < = 60%. All of these species lack SpoIIIAA, SpoIIIAB, SpoIIIF, SpoIIM SpoIIB and GerM (S5 Table in S1 File). A previous work shows that *Sporosarcina* sp. P37 and *Sporosarcina* sp. P33, which belong to the same clade and are similar to other cocci-shaped *Sporosarcina*, lack 38% of the sporulating genes [58]. However, contrary to this work, we do not predict an ortholog for Spo0A in *Sporosarcina* sp. P33. In the case of *Lysinibacillus sphaericus* C3-41 that can form spores [59], sporulation genes such as *bofC*, *spmA*, *spmB*, *sda*, *spoVAA*, *spoVAB*, *spoVID*, *tlp* and *yqfC* involved in several stages of the differentiation process are absent; however, our method identified the product of *spoIIIAD* (SpoIIIAD), which was previously described as absent [29]. In the category nonsporulating-inferred, from the 290 species (Fig 3d), *Eubacterium hallii* EH1 (*Anaerobutyricum hallii* EH1), a species of the class Clostridia, is the only species that presents 10 predicted POs. This includes the Q:AH complex that excludes SpoIIIAB and SpoIIP from the DMP complex. From the 80 species with an unknown phenotype, the predicted POs are represented on average in 37 of the observations (S1 Fig). The work of Abecasis demonstrated that 75% of the genes that form a signature are preserved in endospore formers, which is a feature that is clearly distinguishable from non-endospore formers that have less than 10% the genes preserved [7]. Considering this observation and those presented in this work for the spore formers with experimental evidence, we consider that in our set, 21% (n = 17) of the studied organisms with an unknown phenotype should be spore formers; however, experiments that prove the phenotype should be performed. The list of potential endospore formers is shown in S7 Table in S1 File.

### Missed annotations of the *spoIIIA* operon

The results of the previous section showed that the *spoIIIA* operon tends to be entirely represented in spore formers. We also showed that the organization of the operon *spoIIIA* is an essential trait to identify POs, since it preserves the organization of the genomic context. While inspecting the genomic contexts of *spoIIIA*, we noticed that some intergenic regions are sufficiently sizeable to hold genes. To corroborate if these sizeable regions were a product of incomplete annotations, as described in the Methods section, we performed a BLAST search using BlastX [50], to find missed annotations in 46 endospore formers that lack one or more POs. The predicted proteins were then subjected to a domain search using *hhmscan* to corroborate the presence of the architectures. The procedure yielded 33 proteins whose annotations were missed in the 32 genomes of the 46 tested (see S8 Table in S1 File).

The examination also revealed in five species alternative Pfam domains not considered by our method for the SpoIIIAA protein (PF05621, PF13401, PF13191, and PF13481), since the prediction did not fulfill the proposed selection criteria; this result suggests that a step of manual curation is always necessary to improve the predictions. Remarkably, the new annotated homologs of SpoIIIAA were concentrated in Clostridia. Meanwhile, the homologs of the SpoIIIAE to SpoIIIAH proteins were frequently distributed in Bacilli in different proportions. The SpoIIIAF protein has the highest proportion of homologs (0.93% of the 14 hits were distributed in Bacilli (13 hits), and 0.07% were distributed in Clostridia (one hit)). From the tested set, in *Bacillus* sp. OxB-1, *Jeotgalibacillus malaysiensis* D7, *Lysinibacillus* sp. B2A1, *Lysinibacillus* sp. YS11, *Lysinibacillus sphaericus* C3-41, *Paenisporosarcina*, *Rummeliibacillus stabekisii* PP9, *Solibacillus silvestris* DSM 12223, *Solibacillus silvestris* StLB049, *Sporosarcina psychrophila* DSM 6499, and *Sporosarcina ureae* P32, we could not find the missing homologs of the *spoIIIA* operon.

As in the case of the prediction made using the proposed protein architectures, the RCKO criteria was applied to inspect the upstream or downstream contexts of the blasted sequences. For example, the presence downstream of an acetyl-CoA carboxylase (K01961) from the *spoIIIAH* gene, reinforces the prediction, since this feature is also shared by the 261 other genomes that have SpoIIIAH.

### Signatures beyond representative organisms

The exhaustive examination beyond the representative species may show differences in the phyletic profiles of closely related species. If so, then the observed distribution will generate several questions about the origin and evolution of the sequences that encode the predicted orthologs in the not previous explored species. Therefore, we addressed these two questions, using a curated compendium of experimentally confirmed endospore formers, and the prediction of POs (S5 Table in S1 File). The prediction of the orthologs into a wide set of confirmed phenotypes, is crucial bearing in mind, that several genome projects related to Firmicutes do not prove whether the respective strains can form spores and that several of them infer the phenotype from phylogenetically related strains.

**Fig 4:**
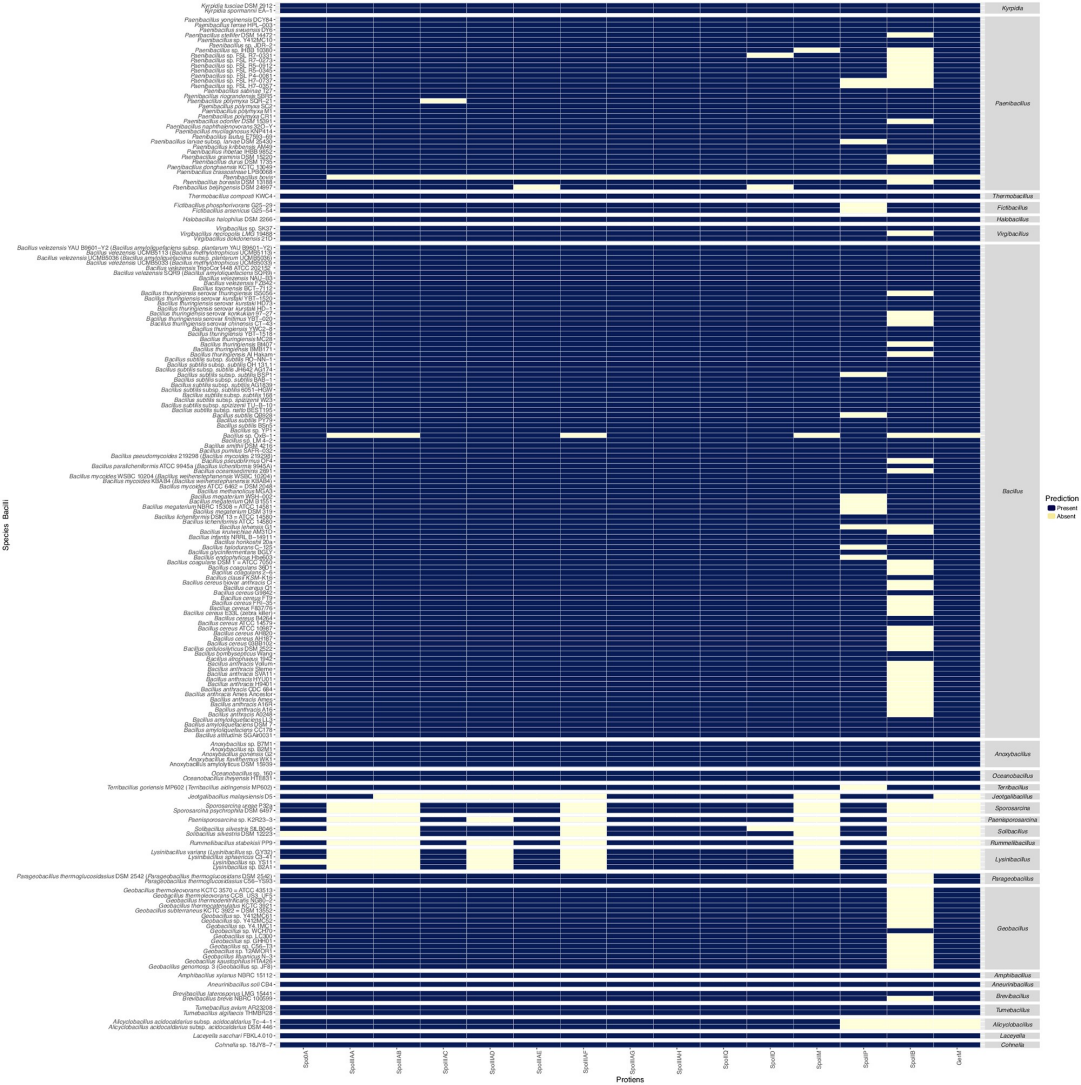
Distribution per genus of the phyletic profiles of sporulating Bacilli. The heatmap presents the predicted phyletic profiles of the predicted orthologs involved in the engulfment of sporulating species from the class Bacilli, grouped by genus.

**Fig 5:**
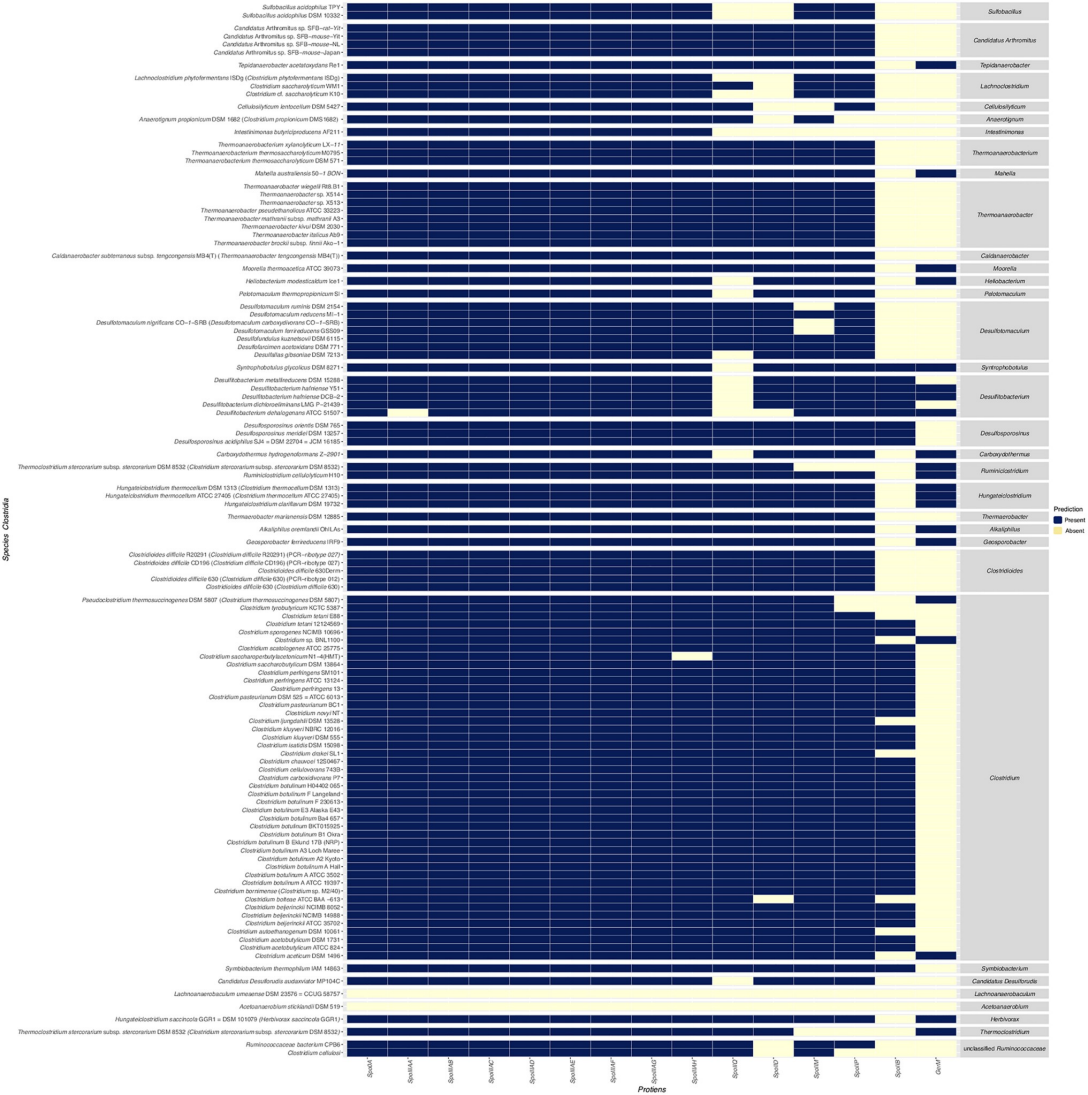
Distribution per genus of the phyletic profiles of sporulating Clostridia. The heatmap presents the predicted phyletic profiles of the predicted orthologs involved in the engulfment of sporulating species from the class Clostridia, grouped by genus.

To evaluate the extent of an endosporulation genomic signature among the species proven to sporulate, we organize each genus by its correspondent class, in order to facilitate an inspection of the results. The organization of the major classes of sporulating Firmicutes (Bacilli and Clostridia), as shown in Figs 4 and 5, indicates that these proteins tend to be preserved into the genus. Despite this, a vast number of them lack at least one PO involved in the engulfment. In 2012, a work of Galperin [29], showed that the Q:AH and the DMP complexes are fully present in sporulating Bacilli, and that particularly the SpoIIIA–SpoIIQ ‘zipper’ should be a common feature of bacillar sporulation. However, the results presented in Fig 4, are more similar to the findings of Ramos-Silva [8], in which the complex is present above 50%. A more precise calculation of the frequency of the representation of the PO per genus of all classes in our results, shows that 98% of the genera preserve at least 50% of the predicted POs. As seen in Fig 5, a search into this wider set found that *Acetoanaerobium* and *Lachnoanaerobaculum*, that have only one species and which belong to Clostridia, are the only genera in which any of the POs was identified. The remaining 31 confirmed endosporulators that have one representative preserve at least 50% of the predicted POs for Bacilli and Clostridia, as seen in Figs 4 and 5.

The remaining 61 genera have at least 2 representatives. The genus *Paenibacillus*, for example, has 34 representatives that stand out for being the only Bacilli in which a species, *Paenibacillus bovis*, none of the tested proteins was detected, despite having Spo0A and a sporulating phenotype [60]. In contrast, as observed in Fig 4, from the 97 species grouped in *Bacillus*, nine do not conserve SpoIIP orthologs, and four of them are strains of *Bacillus megaterium*. Other Bacillus, as seen in Fig 4, also lack SpoIIP, such as those of the genera *Fictibacillus*, *Terribacillus*, and *Alycibacillus*, and four species belong to *Paenibacillus*. The proteins of the DMP complex are also absent among other Bacilli, such as *Jeotgalibacillus*, *Sporosarcina*, *Paenisporosarcina*, *Solibacillus*, *Rummeliibacillus* and *Lysinibacillus*, and all of these consistently lack SpoIIM. The genera organized into Clostridia present similar results as seen in Fig 5; nonetheless, the percentage of the missing POs in the DMP complex, is under 0.9% on average and is concordant with Abecasis’ results [7], which consider the orthologs of these complex as signatures.

The GerM protein, a lipoprotein previously implicated in spore germination, is required to assemble the basal platform of the Q:AH transenvelope complex during sporulation in *B*. *subtilis* [31]. The GerM protein was missing in sixteen species of Bacilli, 95 Clostridia, and the two sporulating Negativicutes. The POs of the GerM in Bacilli were not found in the species of the genera *Jeotgalibacillus*, *Sporosarcina*, *Paenisporosarcina*, *Solibacillus*, *Rummeliibacillus*, and *Alicyclobacillus*. Additionally, only one species of the genera *Bacillus* and *Paenibacillus*, lacked this protein (see Fig 4). In Clostridia, several genera lacked a PO of GerM; this was not the case for all the species that form the genera *Hungateiclostridium* and *Ruminiclostridium*. We observed a PO of GerM in nine genera represented by only one species. No orthologs of GerM are present in *C*. *difficile*. As shown here, a significant number of species of Clostridia and less in Bacilli have an undetected PO. The absence of this protein in such species may imply the existence of a simplified version of the Q:AH complex or that other not yet identified mechanisms will be shaping the engulfasome.

We did not predict the PO of SpoIIB in 78 Bacilli. The POs were also missing in 67 sporulating Clostridia, in two Negativicutes and *Gottschalkia acidurici* 9a, which is classified as a Tissierellia. From Clostridia, the genus *Clostridium* is the more populated; from its 46 species, only nine do not have an identified PO of SpoIIB. Other genera of Clostridia such as *Desulfitobacterium* and *Desulfosporosinus* have more than one representative, and all species possess a predicted PO of SpoIIB. It has been suggested that SpoIIB may have been added late to the engulfment machinery of *B*. *subtilis*, which serves to increase the speed and efficiency of engulfment [32], but this observation may suggest that this protein is perhaps not required by some species to complete engulfment.

Considering this observation, we performed a test to measure the concurrency of the phyletic profile per genus, as described in the Methods section, to determine the prevalence of the studied proteins in the proteomes of the spore formers. The Spo0A response regulator was included in the phyletic profile since it has been mentioned as a sporulation signature of stage 0 in several publications [7, 29, 36, 37], which is stage prior to engulfment. The results in Table 2 show the genus that presents phyletic profiles with a significant C-score in genera that have two or more species. The analysis yielded 28 genera with a defined *phyletic profile signature*. Remarkably, even in the genera that have the largest number of members (*Bacillus* (n = 97), *Clostridium* (n = 46), *Paenibacillus* (n = 34) and *Geobacillus* (n = 16)), a robust signature was identified. The C-score in the genera *Desulfitobacterium* and *Solibacillus* was not significant. The results probably obey to a dissimilarity in the PO distribution observed within *Desulfitobacterium dehalogenans* ATCC 51507, which lacks SpoIIIAA and SpoIID in contrast to the rest of the members of the genus. Two other species, *Desulfitobacterium metallireducens* DSM 15288 and *Desulfitobacterium dichloroeliminans* LMG P–21439, also lack GerM, which affects the distribution across the genus. In contrast, two species grouped as *Solibacillus* (both from the species *silvetris*) lack several POs, which justifies the observed result.

**Table 2:** Prediction of the phyletic profile signatures involved in engulfment per genus based on the phyletic profiles.

Genus	Class	Number of species per class	C-score	Protein group signature
*Bacillus*	Bacilli	97	0.1	Spo0A-Q:AH-DM-GerM
*Clostridium*	Clostridia	46	0.1	Spo0A-Q:AH-DMP-SpoIIB
*Paenibacillus*	Bacilli	34	0.1	Spo0A-Q:AH-DMP-GerM
*Geobacillus*	Bacilli	16	0	Spo0A-Q:AH-DMP-GerM
*Thermoanaerobacter*	Clostridia	8	0	Spo0A-Q:AH-DMP
*Desulfotomaculum*	Clostridia	7	0.1	Spo0A-Q:AH-DMP-SpoIIB
*Anoxybacillus*	Bacilli	5	0	Spo0A-Q:AH-DMP-SpoIIB-GerM
*Clostridioide*	Clostridia	5	0	Spo0A-Q:AH-DMP
*Desulfitobacterium*	Clostridia	5	0.4	Not significant
*Candidatus Arthromitus*	Clostridia	4	0	Spo0A-Q:AH-DMP
*Hungateiclostridium*	Clostridia	4	0	Spo0A-Q:AH-DMP-GerM
*Lysinibacillus*	Bacilli	4	0	SpoIIIC-SpoIIIE-SpoIIIG-SpoIIIH-SpoIIQ-DP
*Desulfosporosinus*	Clostridia	3	0	Spo0A-Q:AH-DMP-SpoIIB
*Lachnoclostridium*	Clostridia	3	0	Spo0A-AH-MP
*Thermoanaerobacterium*	Clostridia	3	0	Spo0A-Q:AH-DMP
*Virgibacillus*	Bacilli	3	0	Spo0A-Q:AH-DMP-GerM
*Alicyclobacillus*	Bacilli	2	0	Spo0A-Q:AH-DM
*Brevibacillus*	Bacilli	2	0	Spo0A-Q:AH-DMP-GerM
*Fictibacillus*	Bacilli	2	0	Spo0A-Q:AH-DM-SpoIIB-GerM
*Kyrpidia*	Bacilli	2	0	Spo0A-Q:AH-DMP-SpoIIB-GerM
*Oceanobacillus*	Bacilli	2	0	Spo0A-Q:AH-DMP-SpoIIB-GerM
*Parageobacillus*	Bacilli	2	0	Spo0A-Q:AH-DMP-GerM
*Pelosinus*	Negativicutes	2	0	Spo0A-Q:AH-DMP
*Ruminiclostridium*	Clostridia	2	0	Spo0A-Q:AH-D-GerM
*Solibacillus*	Bacilli	2	1	Not significant
*Sporosarcina*	Bacilli	2	0	Spo0A-SpoIIIAC-SpoIIIAD-SpoIIIAE-SpoIIIAG-SpoIIIAH-SpoIIQ-DP
*Sulfobacillus*	Clostridia	2	0	Spo0A-AH-MP
*Tepidanaerobacter*	Clostridia	2	0	Spo0A-Q:AH-DMP-GerM
*Tumebacillus*	Bacilli	2	0	Spo0A-Q:AH-DMP-SpoIIB-GerM

The *phyletic profile signatures* are shown for a genus with more than two species. Columns from one to five shows in the following order, the genus grouping the species, the class to which the genus belongs, the number of species per class, and the “Checkerboard score” (C-score) < = 0.2 were considered to be significant, and a C-score > = 0.2 was not considered to be significant; a phyletic profile signature is proposed.

The studied set presents 33 genera defined solely by a species (S9 Table in S1 File) a condition that makes the determination of reliable signatures unfeasible. In this case, the inclusion of new fully sequenced species in each genus will help to proposed phyletic profile signatures.

Finally, to test if the proposed signatures can predict sporulating phenotypes, we tested them on the species with an inferred and unknown phenotype. We found that from the 31 species with a sporulating-inferred phenotype, four do not follow the proposed *phyletic profile signature* (S10 Table in S1 File). The four species belong to different genera (*Bacillus*, *Clostridium*, *Geobacillus*, and *Paenibacillus*). A result suggesting that our signatures are well preserved, and these species do not sporulate. We applied the same procedure to 29 species that belong to the unknown phenotype. We found that in the species of the genera *Bacillus*, *Lachnoclostridium*, *Lysinibacillus Sporosarcina*, *Thermoanaerobacterium* and *Virgibacillus*, the proposed phyletic profile signature was preserved. In the genus *Paenibacillus*, one of the four species (*Paenibacillus* sp. IHB B 3084) has a different phyletic profile organization from that proposed as a signature. For this reason, we predicted a nonsporulating phenotype; in this case, experimental evidence should be collected to verify this result. Additionally, we assign a nonsporulating phenotype to two species of *Ruminiclostridium*, both having distinct phyletic profiles. The signature for *Ruminiclostridium*, was constructed using two species with a reported phenotype; in this case, two species are perhaps not sufficient to establish a signature; however, several species across this genus do not form spores, an observation that suggests that the prediction could be correct. Finally, we observed that *Solibacillus* sp. R5-41 from the genus *Solibacillus* deviates from the proposed pattern since Spo0A is the only missing protein. We decided not to predict a phenotype since the ortholog is the result of a KO prediction. A more accurate prediction should be made to corroborate the presence of the ortholog in the genome.

### Reconstruction of the phylogenetic tree based on the engulfment phyletic profile

The reconstructions of the Firmicutes species trees based on the markers of the 16S gene and ribosomal proteins are often formed by the selection of representative strains. Some of the selected strains are bacterial models that have peculiar phenotypes or traits and biotechnological or medical model organisms. When the biological parameters of selection are not available, the use of a clustering sequence analysis based on their similarities [45], will provide a set of nonredundant sequences that are expected to be able to classify living organisms into taxa that should be consistent with the true phylogenetic tree [61]. Since the phylum Firmicutes is polyphyletic [57] and the species are prone to horizontal gene transfer, the phylum taxonomy is continuously reorganized, especially the Clostridia class [62, 63], which comprises various species of a gram stain-negative cell wall structure [64]. The aforementioned descriptions along with those that claim that the genes related to sporulation have been frequently lost throughout the evolution of Firmicutes [7, 8, 29, 37, 65], motivate to construct a Firmicutes species tree in which the organism selection was based on the organization of the phyletic profiles of the proteins involved in the engulfment and on the curated phenotypes, this means that a genus or a family may have more than one representative. We assume that a selection that uses species that have characteristic phyletic profiles, even if they belong to the same genus or family, will provide new clues that describe the evolutionary history of genes involved in the engulfment.

**Fig 6:**
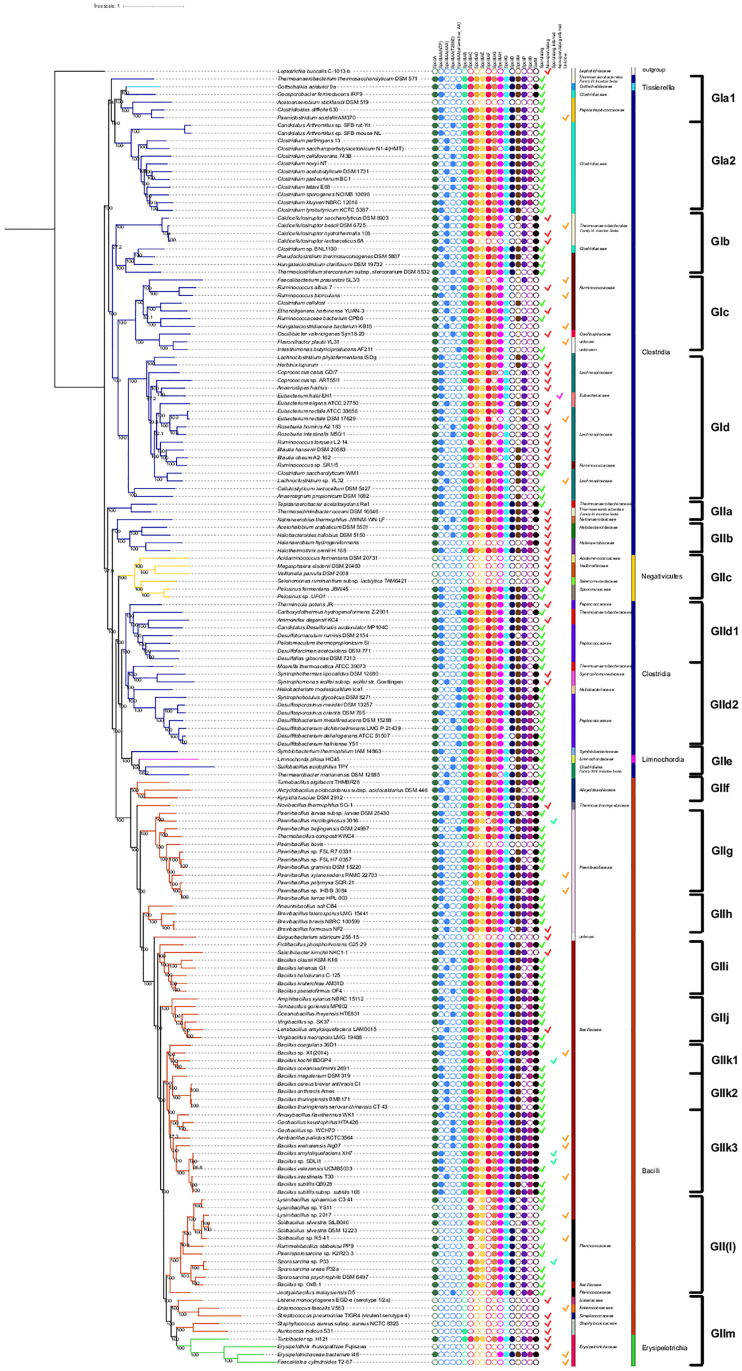
Phylogenetic tree based on a concatenated protein tree and the selection of species by the nonredundant phyletic profiles of the POs involved in engulfment. The species tree is presented next to (right panel) the phyletic profiles and the curated phenotypes. The branches that identify each class and family appear in different colors next to the tree. For the SpoIIIAA proteins, the predicted architecture of each organism is also shown.

To test this hypothesis, we concatenated 585 proteins preserved in 90% of the species to construct a species tree. This final tree was then compared with some previously proposed Firmicutes trees [8, 57, 62–64]. Our final tree clustered 167 Firmicutes including 99 sporulating species, 5 species with an inferred sporulating phenotype, 44 nonsporulating species, 19 species with an unknown phenotype, and one outgroup. The phyletic profile associated with each species is represented next to the tree as shown in Fig 6. The predictions calculated for Spo0A for 259 Firmicutes [36] and complemented with the genes related to K07699 assigned by KEGG to Spo0A were included in the represented phyletic profile. These additions were made to ensure the presence of a protein considered to be a signature involved in the preceding stage of sporulation.

As seen in Fig 6, the tree is well resolved at almost all nodes, in which as previously reported [8, 37, 63], the classes Bacilli (n = 71) and Clostridia (n = 87) are separated. The analysis yielded a first split that separates Clostridia into the two groups GI and GII. GI forms four clusters that harbor members of the families *Leptotrichiaceae*, *Thermoanaerobacterales Family III*. *Incertae Sedis*, *Gottschalkiaceae*, *Clostridiaceae*, *Peptostreptococcacea*, *Ruminococcaceae*, *Oscillospiraceae*, *Lachnospiraceae*, *Eubacteriaceae*, and two unknown families. The tips resolution shows the species consistently clustered, except for *Thermoanaerobacterium thermosaccharolyticum* DSM 571, *Clostridium* sp. BNL1100, *Geosporobacter ferrireducens* IRF9, and *Clostridium saccharolyticum* WM1, which appeared in branches out of the reported family. GIa is divided into two clusters, the first one, GIa1 including *Thermoanaerobacterium thermosaccharolyticum* DSM 571, *Gottschalkia acidurici* 9a a member of the class Tissierellia, class, which as reported forms a cluster with the members of *Clostridiaceae* and *Peptostreptococcacea* [62], and all of them lacking SpoIIB. *Geosporobacter ferrireducens* IRF9 that branches with *Gottschalkia acidurici* 9a share the presence of the POs of GerM, as opposed to the rest of the members within the clade. *Acetoanaerobium sticklandii* DSM 519 is also grouped in GIa1, which was recently transferred to the *Peptostreptococcacea* family [66]. Despite being reported as a spore former (see S5 Table in S1 File) [67], our analysis shows that this bacteria preserves neither the genes that encode the engulfasome and the accessory proteins nor Spo0A, a trait seen in some Firmicutes, an example is *Limnochorda pilosa* a genome that lost the genes involved in sporulation although they have been regarded as essential for the endospore forming system [68]. A second branching grouped the family *Clostridiaceae*, which all form spores and lack GerM but preserve the rest of the studied proteins, except for *Clostridium tetani* E88 for which we did not find a PO for SpoIIB.

GIb holds two groups of Firmicutes, a set of nonsporulating species of family *Thermoanaerobacterales Family III*. *Incertae Sedis*, which is represented by a group of *Caldicellulosiruptor* that share a last common ancestor with a branch of sporulating *Ruminococcaceae* and *Clostridium* sp. BNL1100. In contrast to what is reported [69], and looking at the bootstrap value (27.2%), this cluster is probably not well resolved. However, the internal branches that grouped *Hungateiclostridium clariflavum* DSM 19732 and *Thermoclostridium stercorarium* subsp. *stercorarium* DSM 8532, are consistent, with previous reports. These species previously named *Clostridium*, are subject to fast evolution [65], which may explain the bootstrap value and missed locations of *Thermoanaerobacterium thermosaccharolyticum* DSM 571, placed near the root.

GIc harbors two groups of *Ruminococcaceae* of the genus *Ruminococcus*, *Faecalibacterium* and *Ruminiclostridiu*, an *Oscillospiraceae* and two species with an unknown family. This cluster displays an important number of species with an unknown phenotype characterized by preserving the POs of the *spoIIIA* operon except for *Flavonifractor plautii* YL31, that lacks POs of the SpoIIQ, SpoIIB, GerM proteins and the POs of the DMP complex. Notably, all the species in this group lack SpoIID and SpoIIB, and the rest of the proteins of the DMP complex are almost missing in all species but are better preserved in the sporulating ones.

GId which groups the species from the families *Lachnospiraceae and Eubacteriaceae* (n = 19) shared a common ancestor; however, two endospore formers, specifically, *Cellulosilyticum lentocellum* DSM 5427 and *Anaerotignum propionicum* DSM 1682, are grouped separately from a larger clade that clustered two *Eubacteriaceae (Eubacterium hallii* EH1 and *Eubacterium eligens* ATCC 27750), previously reported to be grouped within *Lachnospiraceae* [70, 71]. Twelve of the species clustered within this clade are not endospore formers; nonetheless, most of them have preserved POs of the *spoIIIA* operon and all conserve a PO of SpoIIP, but they lack POs of SpoIID, SpoIIB and GerM. Remarkably, the nonsporulating species were also split from the sporulating ones, which holds *Eubacterium rectale* DSM 17629 that has an unknown phenotype but a different phyletic profile.

The second split, GII, shows several divisions that harbor a group of Negativicutes and the families from the order Halanaerobiales (n = 4), which belong to the classically monoderm Firmicutes that possess outer membranes with lipopolysaccharide [64]. GIIa, which groups *Tepidanaerobacter acetatoxydans* Re1 and *Thermosediminibacter oceani* DSM 16646 belong to different families. The families *Natranaerobiaceae*, *Halobacteroidaceae* and *Halanaerobiaceae*, grouped in GIIb, are consistent with previous reports [6, 64]. All of the species do not form spores, despite they have POs of the DMP and Q:AH complexes. The members of families *Halobacteroidaceae* and *Halanaerobiaceae* are diderm bacteria with a gram negative-type cell envelope a characteristic shared with the families that belong to the class Negativicutes and Limnochordia [64, 72]. A recent phylogenetic bacterial reconstruction based on outer membrane proteins, shows that these families shared a diderm common ancestor [72]. Our tree reconstruction agrees with the reconstruction made with the just mentioned work except for *Natranaerobius thermophilus* JW/NM-WN-LF that was grouped with uncultured species of bacteria and archaea.

Consistent with other reports the class Negativicutes, (n = 6), is branched within the class Clostridia [6, 63, 64], in group GIIc. The Negativicutes are branched in clades that separate sporulating from nonsporulating species for which any PO was predicted. As seen in reported reconstructions, the proposed trees and the one reconstructed in this work relate the Negativicutes to the families of *Peptococcaceae* and *Thermoanaerobacteraceae* (GIId) [6, 63, 64]. GIId also holds species of the families *Syntrophomonadaceae* and *Heliobacteriaceae*, which are reported to cluster with *Peptococcaceae* and *Thermoanaerobacteraceae* [69]. GIId split *Peptococcaceae* into two groups, GIId1, in which all the species lack the POs of SpoIIB and GerM except for nonsporulating *Thermincola potens* JR. GIId2 housing the second group of *Peptococcaceae* and the families *Thermoanaerobacteraceae*, *Heliobacteriaceae*, and *Syntrophomonadaceae*. The two last ones present the same POs organization patterns. All the *Peptococcaceae* in this branch lack GerM, not being the case of *Desulfosporosinus meridiei* DSM 13257 and *Desulfitobacterium hafniense* Y51.

GIe clustered *Limnochorda pilosa* HC45T, a gram-negative stained bacteria that represents the family *Limnochordaceae*, and this strain is clustered with *Thermaerobacter marianensis* DSM 12885T, *Sulfobacillus acidophilus* DSM 10332T and *Symbiobacterium thermophilum* IAM 14863T, which have a high GC content except for *Sulfobacillus acidophilus* TPY that has a lower GC content [68]. The four species are spore formers. Several sporulation genes are absent in *Limnochorda pilosa* [68], for which we observed only the absence of a PO of SpoIID. For its part, *Sulfobacillus* also lacks a PO of SpoIID and SpoIIB, which is a characteristic shared with *Thermaerobacter* that also lacks the POs of GerM. All of the species of this group except for *Thermaerobacter* have a complete version of the *spoIIIA* operon for which SpoIIIAH is missing in this species. In this cluster, SpoIIQ is missing only in *Sulfobacillus acidophilus* TPY.

The class Bacilli contains nine families that are organized in our tree in seven groups. GIIf clustered the spore formers of the family *Alicyclobacillaceae* in which *Alicyclobacillus acidocaldarius* subsp. *acidocaldarius* DSM 446 has undetected POs of SpoIIP, SpoIIB and GerM, as opposed to the rest of the members of the group that have a complete set of the studied proteins. This result contrasts with another report in which the orthologs of SpoIIP and SpoIIB were not found in *Kyrpides tuscia* DSM 2912 [29]. The next division shows *Novibacillus thermophilus* SG-1 located as a singleton within Bacilli in the family *Thermoactinomycetaceae*; in another report, this species group with another strain that forms a separate node from the rest of the *Thermoactinomycetaceae* [73], and in this nonspore former, we were not able to detect the POs of SpoIIIAA and SpoIID.

The family *Paenibacillaceae* appeared in our tree as divided into two branches, and GIIg grouped the species of the genera *Paenibacillus* and *Thermobacillus* in the same branch consistent with previous reports [8, 69, 74]. In this group, *Paenibacillus bovis* is a spore former that lacks all the studied proteins. A study in which the conservation of modularity was inspected in several Firmicutes showed that *Paenibacillus bovis* has a relative low conservation to the average of the sporulation genes across the analyzed species [74]. GIIh grouped *Paenibacillaceae* of the genus *Brevibacillus* and *Aneurinibacillus* in this cluster, and the nonsporulating *Brevibacillus formosus* NF2 that lacks the POs of SpoIIB and SpoIID.

Within GII, *Exiguobacterium sibiricum* 255–15 as *Paenibacillus bovis* is another species for which the analysis does not reveal the POs of the studied proteins, and similar to *Paenibacillus bovis*, the study based on modularity showed a lower conservation of genes depending on σ^E^ and σ^F^ expressions [74], that as mentioned these sigma factors control genes that are involved the engulfment in Bacilli and Clostridia. In this GII mega-cluster, the families *Planococcaceae* and *Bacillaceae* have several genera that form independent clusters. GIIi holds species of the family *Bacillaceae* that mainly lack the orthologs of SpoIIP and SpoIIB. GIIj preset *Lentibacillus amyloliquefaciens* LAM0015, a nonspore former that encodes the POs of all the proteins in its genome but lacks Spo0A, which probably prevents the onset of sporulation. In GIIj, we also found sporulating *Bacillaceae* from the genera *Virgibacillus* and *Oceanobacillus* in which *Virgibacillus necropolis* LMG 19488 lacks SpoIIB. We found in GIIk 16 species of *Bacillus* (GkII1,2,3), two *Geobacillus* (GIIk3) and one of *Anoxybacillus* (*Anoxybacillus flavithermus* WK1) (GIIk3), which are consistently grouped [69].

GII(I) group members of the families *Bacillaceae* and *Planococcaceae* have a branch that clusters congruently with the genus *Lysinibacillus* (n = 3) and *Solibacillus* (n = 3), respectively [69]. *Lysinibacillus* is known to be highly polyphyletic [75], which is a feature preserved in our reconstructed tree. Other genera such as *Rummeliibacillus*, *Paenisporosarcina* and *Sporosarcina* are congruently resolved [75]. The *Sporosarcina* clade includes within species from the genus *Sporosarcina* along with *Bacillus* sp. OxB-1 [75]. Despite forming spores, all the members lack several genes that encode the POs of the *spoIIIA* operon (SpoIIIAA, SpoIIIAB and SpoIIIAF in all species and SpoIIIAD in five species) and all the POs of SpoIIM, SpoIIB and GerM. It is remarkable the absence of Spo0A in *Sporosarcina* sp. P33, for which the KEGG database did not assigned a KO, and we did not test by our method [36], so an update of our data will corroborate this result. *Jeotgalibacillus malaysiensis* D5 possess POs of SpoIIIAA and SpoIIB, this is not the case of SpoIIIAC and SpoIIIAE, which are missing in the profile, a result that contrasts with the rest of the observations within the group.

As seen, GIIm clusters a group of species that belongs to the families chosen because all the species are nonsporulating, except for *Auricoccus indicus* S3 in which SpoIIIAB was predicted, indicating that maybe the gene that encodes this protein was gain as a consequence of the adaptive forces prevalent in the phylum. The species of the class Erysipelotrichia (n = 4), are consistently branched near *Staphylococcaceae* [8, 69], but the omission of the *Tenericutes* (*Mollicutes*) species in our tree prevents seeing the remote relationship between Erysipelotrichia and *Mollicutes*. *Erysipelotrichaceae bacterium* I46 does not form spores, and *Turicibacter* sp. H121 has an unknown phenotype; they preserved the POs mainly related to the DMP complex, SpoIIB, GerM, and Spo0A but lack most of the POs encoded in *spoIIIA*, suggesting that the absence of these gene products and the sigma factors regulating their expression [8] underline the versatility of strategies to adapt to different environment.

### Gain and loss events are common in the proteins involved in the engulfment

**Fig 7:**
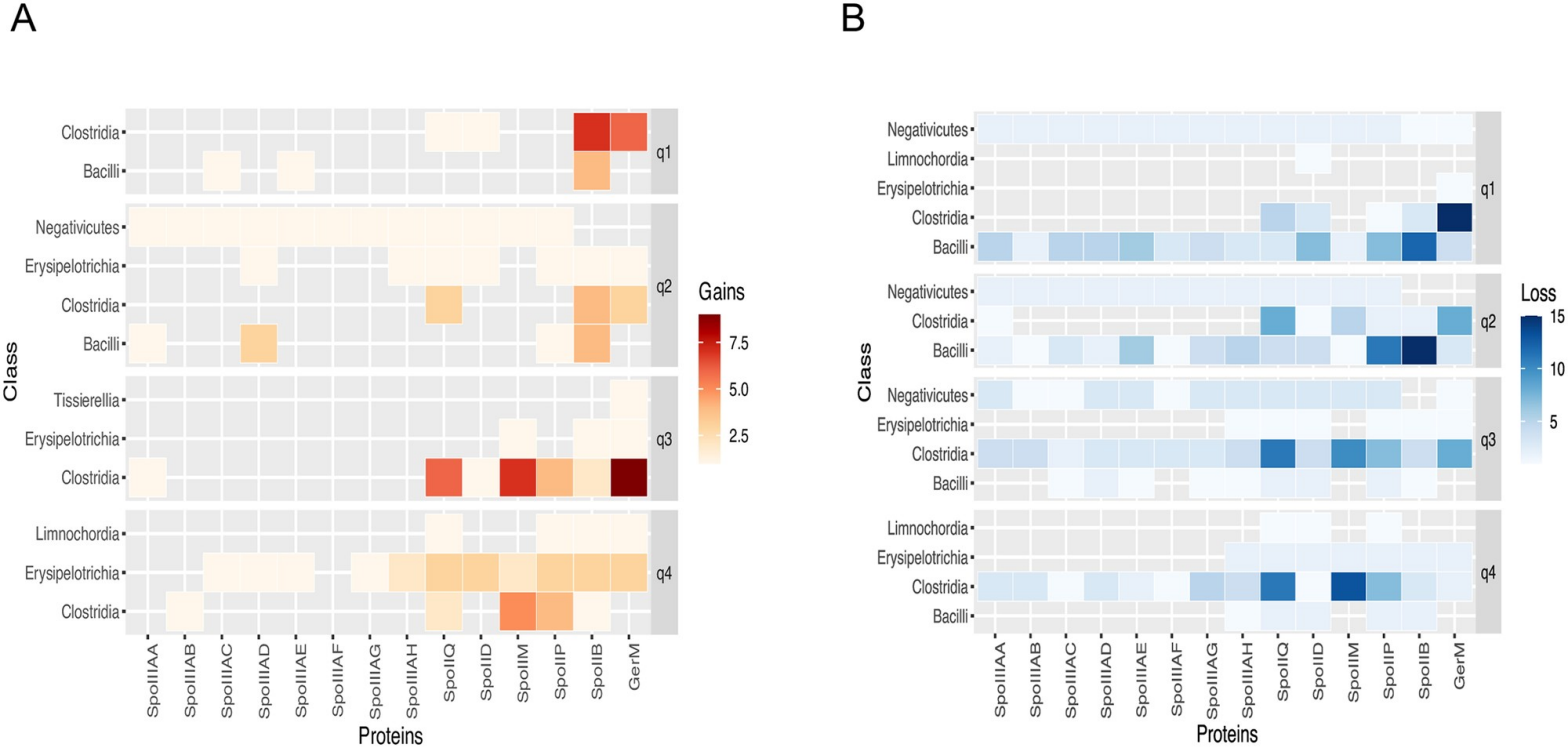
Expected number of the gene gain and loss events for each protein. The posterior expectation for the gain (A) and loss (B) events was estimated for each protein involved in engulfment on each branch of the species tree.

The patchy distribution observed in the phyletic patterns across the 954 Firmicutes and the prediction of orthologs in previous works [7–9, 29, 65], show that the sporulation genes, even those defined as signatures, may be missing in some species. Several authors postulated that horizontal gene transfer and loss events may be important evolutionary forces of the sporulation genes among Firmicutes. Therefore, we also sought to analyze the expected number of the gene gain and loss events of the proteins involved in engulfment across the species used to reconstruct the phylogenetic tree by using GLOOME, which is a tool that provides accurate estimates of the expectations and probabilities of both gains and losses [54]. GLOOME infers the position of the gene gain and loss events across a phylogenetic tree but does not consider duplication and speciation events. To evaluate the GLOOME results, the distance from the root to the tips was distributed in quantiles (q_n_). The first quantile (q1) represents the nodes that have the shortest distances from the root, and q4 represents the nodes with positions frequently on the tips (S11 Table in S1 File). As a result, we found that the nodes and branches distributed in q_1_ have 133 gains and 441 loss events, with a major loss event of GerM in Clostridia (q1), and as seen in Fig 7b, the loss of GerM in Clostridia seems to occurred in the last common ancestor of the group, but a set of few gains appeared in q1, less in q2, and several gains in q3, which can be seen in Fig 7a. These gains were preserved among most of the sporulating Bacilli.

An important gain event of SpoIIB can be seen in Clostridia (q1), as also some gains in Bacilli in q1 and q2. The results suggest that these gains were acquired after a major loss event that occurred twice in nodes placed in q1 a q2. A minor loss of SpoIIP is observed, in the nodes and branches distributed in q1 also in Bacilli. The orthologs of SpoIIM were also frequently lost in Clostridia in the branches grouped in q3 and q4 but not in q1, which suggests that this protein was present in the last common ancestor of the species within this class similarly to the POs of the genes encoded in the *spoIIIA* operon, which were lost more recently (q3 and q4). SpoIIQ had several losses across the tree that were more frequent in the branches positioned in q3 and q4 in species related to Clostridia that are located in the GII branch of the tree. Notably, the gains observed in Negativicutes in q2 were then lost in the nonsporulating species in the nodes and branches distributed in q3. The loss events found in Erysipelotrichia were seen in ancestral branches located in q2 that began in the asporogenous lineages of the class Bacilli (*Listeriaceae*) with an important gain (q4) in Erysipelotrichia in all of the genes encoding the studied proteins except for SpoIIIAA, SpoIIIAB and SpoIIIAF for which the event was not observed, a result that is consistent with that found in a previous work [8]. Important losses of the SpoIIIAA, SpoIIIAB, SpoIIIAD and SpoIIIAF were also seen in Bacillus in q1, this losses correspond to species of the family *Planococcaceae* and some species of the genus *Lysinibacillus* in which several of them form spores. The results found in *Planococcaceae* are concordant with a previous report that shows an extensive loss in the genus *Solibacillus* [8], which are extended in this work to other *Planococcaceae* (Fig 6). In the same family, the sporulating species *Jeotgalibacillus malaysiensis* D5, presents in q2 a gain of SpoIIIAA and the loss of SpoIIIAC and SpoIIIAE; this result shows the need for experimental studies in Firmicutes other than *C*. *difficile* and *B*. *subtilis*, which explain the mechanisms used by these organisms to carry out the sporulation process.

## Discussion

### Components of engulfment beyond representatives

The analysis conducted in this work based on the similarity of the proteins involved in engulfment and the proteins SpoIIB and GerM showed that the distribution of these proteins varied among the 954 studied Firmicutes, even in the species that produce spores. These deviations have been better studied in *B*. *subtilis* and *C*. *difficile* whose experimental evidence shows differences in the regulation and mechanisms and in the genes needed to promote engulfment [1, 18, 19]. The differences were also found in other stages of sporulation that have been the object of study by groups that, given the lack of or little experimental evidence in other species, have been motivated to search for these differences by sequences comparison, and some of them have found genes that serve as sporulation markers or signatures [5, 7–9, 29, 76]. However, the prevalence of sporulation markers among the “asporogenic” species of Firmicutes and the absence of some of them in sporulating species generate important questions regarding their evolution in the phylum. Particularly, in the cases of the genes involved in engulfment, several studies have identified the products of the *spoIIIA* operon as part of the core genes conserved in endospore formers [5, 7–9, 29, 76]. Our findings that consider 954 Firmicutes show that the genes of the operon prevail in the highest proportion of endospore formers in contrast to the genes that do not form spores, which is a result shown in other reports. In a similar work conducted with 258 representative Firmicutes [8], the authors showed the absence of SpoIIIAF in the genera *Paenibacillus* (n = 4), *Thermobacillus* (n = 1) and in *Solibacillus silvestris* StLB046. Using a wider data set and our developed methodological approach, we found differences between our results and those presented in this similar work. For these aforementioned genera, we predicted the POs of SpoIIIAF in eleven tested *Paenibacillus* and in one *Thermobacillus* and an agreement with the absence of SpoIIAF in *Solibacillus silvestris* StLB046. We also found that the *Solibacillus* species included in our study and other members of the family *Planococcaceae* also lack the POs of this protein. Remarkably, other absent orthologs of the *spoIIIA* operon in *Planococcaceae* were SpoIIIA-B and SpoIIIAG; SpoIIIAG was searched with the HMM profile constructed in this work, which efficiently recovered the SpoIIIAG orthologs. In *B*. *subtilis*, it is suggested that the role of the proteins encoded in the *spoIIIA* operon sustain transcription activity of σ^G^ in the forespore [25], so the mechanism used to promote the engulfment by the analyzed *Planococcaceae* and the members of the genus *Lysinibacillus* remains to be elucidated.

The orthologous genes that encode SpoIIQ and the proteins of the DMP complex are better conserved in sporulating Bacilli and Clostridia, and the frequencies are lower than those found for the PO of *spoIIIA*, especially in Clostridia. These results suggest that the mechanisms proposed for the engulfment progression may be different in some of the analyzed species, as for example the genera *Sulfobacillus* and *Lachnoclostridium*, which lack POs of SpoIIQ and SpoIID, which in *C*. *difficile* were proven to have strong interaction, however, some cells carrying the *spoIID* deletion seem to be able to partially initiate engulfment [27].

The finding in 24 representatives that was recently reported [9], shows the absence of the orthologs of SpoIIP and SpoIIM in only two species, namely, *Symbiobacterium thermophilum* and *Lysinibacillus sphaericus*, respectively. Our study detected the POs of DMP in *Symbiobacterium thermophilum*, but the results for *Lysinibacillus sphaericus* prevail in this species as in other two analyzed *Lysinibacillus*. The observed results may lead to a hypothesis that the same selection pressures affected the patterns observed for the complex in these species. Comparisons of our extended search with the results found in other works that use a limited number of species to represent each genus showed that the phyletic patterns may change within clades, and as discussed in other studies [7, 8, 29], a few sets of genes that participate in this complex differentiation process can be considered to be permanent signatures. Moreover, the preservation of the sporulation genes in asporogenic species still make it difficult to infer that a species is an endospore former solely based on the presence of the so-called signatures.

### Signature based on phyletic profile

The employed approach that uses HMM profiles and the preservation of the RCKOs are powerful tools to predict distant orthologs, as they allow the extension of the number of POs across a wider set, and we predicted orthologs not found using other methodologies. Moreover, the careful inspection of the genomic context was fundamental to identifying missed annotated genes that belong to the *spoIIIA* operon and showed that the genes of the *spoIIIA* operon were preserved on average in 97% of the endospore formers, which confirms that these proteins and the proteins of the DMP complex were also preserved on average in 97% of the endospore formers and are, as previously described, sporulation signatures [7, 29]. This observation is more consistent for the species in the class Bacilli, however as other authors discussed these genes as others define as signature are not a strong trace allowing to infer the sporulation phenotype in Firmicutes. Despite this, we used the distribution of these signatures into each genus to estimate the probability that a given phyletic profile can function as a signature of this sporulation stage, finding a set of proteins prevailing for each genus, that were then used to predict a sporulation phenotype in species for which the experimental evidence does not exist. The constructed phyletic profile signatures as shown, were in almost all of the tested genus able to predict a phenotype, a result that should be experimentally proven. Unfortunately, for 33 genera that have an only member a dependable signature was impossible to predict, we are confident that the exploration of new environments, will provide new Firmicutes that may help to refine the proposed signatures and get some for the species that in this work were represented by an only organism.

### Evolution of the protein involved in the engulfment based on phyletic profiles

Tree reconstruction and the estimation of gain and loss events, in our study were based on the selection of species having unique phyletic profiles within families. As seen in other reconstructions the selection of species conforming the tree and the genes chosen gives some differences in the arrangement of some branches, a trait seen in our results. This does not prevent us to see that in the selected species representing each family, the observed patterns reflect particular changes that have been preserved among their members, being more evident among those species sharing a common origin, a result showed in other woks [7, 8, 37]. In these work and ours, some of the proteins in particular those involved in the engulfasome and the accessory proteins GerM and SpoIIB are confined to the phylum Firmicutes, letting trace the evolution of the engulfment proteins at the divergence of Firmicutes from other prokaryotic phyla, estimated to have occurred somewhere around 2.5 to 3.0 million years ago [77]. Moreover, the aforementioned studies, in which the sporulation proteins were grouped considering the sigma factor affecting their gene regulation, did not trace the evolution of these sigma in which some of them as σ^E^ and σ^G^ affect the regulation of genes involved in functions different from sporulation [78], which will give other clues about the origin and evolution of the different strategies taken by this phylum to adapt to different environments. The loss and gain analysis performed by the GLOOME program only identifies the horizontal gene transfer (HGT) events that result in the gain of the studied proteins, and we cannot be certain whether the coding genes were gained by HGT or gene duplication; however, the results found in this analysis shows consistent results as those found in previous works. Finally, the gain events were seen at the base of the tree for the protein SpoIIB in Clostridia and a posterior loss in some members of Bacilli, a result suggesting that as previously tested in *B*. *subtilis*, a mutant in the gene *spoIIB* can complete engulfment, that probably causes a defect affecting only the speed of engulfment [32].

## Conclusions

In this study we inspected the proteomes of 954 Firmicutes, to identify POs of a set of proteins involved in the engulfment. The performed study included a wide number of species, showing for the first time the distribution of this component in a significant number of species, showing phyletic profiles that prevail into genus, and that we proposed to be used as sporulation signatures. The patterns were tested over Firmicutes with an unknown phenotype for which we proposed that this signature can be used to suggest a sporulating or a nonsporulating phenotype. We think that this first attempt should be complemented with proteins participating in other stages of sporulation, which can better support the predicted phenotype. We also showed a phylogenetic tree in which the displayed species were selected using unique phyletic profiles across taxonomic families, which yield a consistent reconstruction revealing that the genes encoding proteins involved in the engulfment was present in the last common ancestor of this phylum, with subsequent losses and gains that occurred in different points along the phylogeny.

## Supporting information

S1 File Supplementary tables. (XLSX)

S1 Fig (PDF)

## Abbreviations

*B. subtilis*: *Bacillus subtilis*
*C. difficile*: *Clostridioides difficile*
C-score: Checkerboard score
HMM: hidden Markov model
KO: KEGG ortholog
PO: probable ortholog
RCKO: representative contextual KEGG ortholog

## References

[1] Al-Hinai MA, Jones SW, Papoutsakis ET. The Clostridium Sporulation Programs: Diversity and Preservation of Endospore Differentiation. Microbiol Mol Biol Rev. 2015;79:19–37. Available from: http://mmbr.asm.org/lookup/doi/10.1128/MMBR.00025-14 25631287 10.1128/MMBR.00025-14 PMC4402964

[2] Fimlaid KA, Bond JP, Schutz KC, Putnam EE, Leung JM, Lawley TD, et al. Global Analysis of the Sporulation Pathway of Clostridium difficile. PLoS Genet. 2013;9. 10.1371/journal.pgen.1003660 23950727 PMC3738446

[3] Hutchison EA, Miller DA, Angert ER. Sporulation in Bacteria: Beyond the Standard Model. Microbiol Spectr. 2014;2:1–15. 10.1128/microbiolspec.TBS-0013-2012 26104376

[4] Higgins D, Dworkin J. Recent progress in Bacillus subtilis sporulation. FEMS Microbiol Rev. 2012;36:131–48. 10.1111/j.1574-6976.2011.00310.x 22091839 PMC3237856

[5] Onyenwoke RU, Brill JA, Farahi K, Wiegel J. Sporulation genes in members of the low G+C Gram-type-positive phylogenetic branch (Firmicutes). Arch Microbiol. 2004;182:182–92. 10.1007/s00203-004-0696-y 15340788

[6] Galperin MY. Genome Diversity of Spore-Forming Firmicutes. Microbiol Spectr. 2013;1:1–27. Available from: http://www.asmscience.org/content/journal/microbiolspec/10.1128/microbiolspectrum.TBS-0015-2012 26184964 10.1128/microbiolspectrum.TBS-0015-2012 PMC4306282

[7] Abecasis AB, Serrano M, Alves R, Quintais L, Pereira-Leal JB, Henriques AO. A genomic signature and the identification of new sporulation genes. J Bacteriol. 2013;195:2101–15. 10.1128/JB.02110-12 23396918 PMC3624599

[8] Ramos-Silva P, Serrano M, Henriques AO. From Root to Tips: Sporulation Evolution and Specialization in Bacillus subtilis and the Intestinal Pathogen Clostridioides difficile. Mol Biol Evol. 2019;36:2714–36. 10.1093/molbev/msz175 31350897 PMC6878958

[9] Kelly A, Salgado PS. The engulfasome in C. difficile: Variations on protein machineries. Anaerobe. 2019;60:102091. 10.1016/j.anaerobe.2019.102091 31470088 PMC6934232

[10] Browne HP, Forster SC, Anonye BO, Kumar N, Neville BA, Stares MD, et al. Culturing of “unculturable” human microbiota reveals novel taxa and extensive sporulation. Nature. Nature Publishing Group; 2016;533:543–6. Available from: http://www.nature.com/doifinder/10.1038/nature17645 27144353 10.1038/nature17645 PMC4890681

[11] Phillips ZE V, Strauch MA. Bacillus subtilis sporulation and stationary phase gene expression. Cell Mol Life Sci. 2002;59:392–402. 10.1007/s00018-002-8431-9 11964117 PMC11337539

[12] Alsaker K V, Papoutsakis ET. Transcriptional Program of Early Sporulation and Stationary-Phase Events in Clostridium acetobutylicum †. J Bacteriol. 2005;187:7103–18. 10.1128/JB.187.20.7103-7118.2005 16199581 PMC1251621

[13] Piggot PJ. Spore development in Bacillus subtilis. Curr Opin Genet Dev. 1996;6:531–7. 10.1016/s0959-437x(96)80080-3 8939730

[14] Chastanet A, Losick R. Engulfment during sporulation in Bacillus subtilis is governed by a multi-protein complex containing tandemly acting autolysins. Mol Microbiol. 2007 [cited 2014 Dec 22];64:139–52. Available from: http://www.ncbi.nlm.nih.gov/pubmed/17376078 10.1111/j.1365-2958.2007.05652.x 17376078

[15] Morlot C, Uehara T, Marquis KA, Bernhardt TG, Rudner DZ. A highly coordinated cell wall degradation machine governs spore morphogenesis in Bacillus subtilis. Genes Dev. 2010;24:411–22. 10.1101/gad.1878110 20159959 PMC2816739

[16] Jones SW, Paredes CJ, Tracy B, Cheng N, Sillers R, Senger RS, et al. The transcriptional program underlying the physiology of clostridial sporulation. Genome Biol. 2008;9. 10.1186/gb-2008-9-7-r114 18631379 PMC2530871

[17] Paredes CJ, Alsaker K V., Papoutsakis ET. A comparative genomic view of clostridial sporulation and physiology. Nat Rev Microbiol. 2005;3:969–78. 10.1038/nrmicro1288 16261177

[18] Saujet L, Pereira FC, Henriques AO, Martin-Verstraete I. The regulatory network controlling spore formation in Clostridium difficile. FEMS Microbiol Lett. 2014;358:1–10. 10.1111/1574-6968.12540 25048412

[19] Pereira FC, Saujet L, Tomé AR, Serrano M, Monot M, Couture-Tosi E, et al. The Spore Differentiation Pathway in the Enteric Pathogen Clostridium difficile. PLoS Genet. 2013;9. 10.1371/journal.pgen.1003782 24098139 PMC3789829

[20] Ribis JW, Fimlaid KA, Shen A. Differential requirements for conserved peptidoglycan remodeling enzymes during Clostridioides difficile spore formation. Mol Microbiol. 2018;110:370–89. 10.1111/mmi.14090 30066347 PMC6311989

[21] Morlot C, Rodrigues CDA. The New Kid on the Block: A Specialized Secretion System during Bacterial Sporulation. Trends Microbiol. Elsevier Ltd; 2018;26:663–76. Available from: 10.1016/j.tim.2018.01.001 29475625

[22] Zeytuni N, Strynadka NCJ. A Hybrid Secretion System Facilitates Bacterial Sporulation: A Structural Perspective. Protein Secret Bact. 2019;389–99. 10.1128/microbiolspec.PSIB-0013-2018 30681070 PMC11588154

[23] Zeytuni N, Flanagan KA, Worrall LJ, Massoni SC, Camp AH, Strynadka NCJ. Structural characterization of SpoIIIAB sporulation-essential protein in Bacillus subtilis. J Struct Biol. Elsevier; 2018;202:105–12. Available from: 10.1016/j.jsb.2017.12.009 29288127 PMC5864543

[24] Zeytuni N, Flanagan KA, Worrall LJ, Massoni SC, Camp AH, Strynadka NCJ. Structural and biochemical characterization of SpoIIIAF, a component of a sporulation-essential channel in Bacillus subtilis. J Struct Biol. Elsevier; 2018;204:1–8. 10.1016/j.jsb.2018.06.002 29886194

[25] Doan T, Morlot C, Meisner J, Serrano M, Henriques AO, Moran CP, et al. Novel secretion apparatus maintains spore integrity and developmental gene expression in Bacillus subtilis. PLoS Genet. 2009;5. 10.1371/journal.pgen.1000566 19609349 PMC2703783

[26] Camp AH, Losick R. A novel pathway of intercellular signalling in Bacillus subtilis involves a protein with similarity to a component of type III secretion channels. Mol Microbiol. 2008;69:402–17. 10.1111/j.1365-2958.2008.06289.x 18485064 PMC2574792

[27] Dembek M, Kelly A, Barwinska-Sendra A, Tarrant E, Stanley WA, Vollmer D, et al. Peptidoglycan degradation machinery in Clostridium difficile forespore engulfment. Mol Microbiol. 2018;110:390–410. 10.1111/mmi.14091 30066424 PMC6221140

[28] Fimlaid KA, Jensen O, Donnelly ML, Siegrist MS, Shen A. Regulation of Clostridium difficile Spore Formation by the SpoIIQ and SpoIIIA Proteins. PLoS Genet. 2015;11:1–35. 10.1371/journal.pgen.1005562 26465937 PMC4605598

[29] Galperin MY, Mekhedov SL, Puigbo P, Smirnov S, Wolf YI, Rigden DJ. Genomic determinants of sporulation in Bacilli and Clostridia: Towards the minimal set of sporulation-specific genes. Environ Microbiol. 2012;14:2870–90. 10.1111/j.1462-2920.2012.02841.x 22882546 PMC3533761

[30] Meisner J, Wang X, Serrano M, Henriques AO, Moran CP. A channel connecting the mother cell and forespore during bacterial endospore formation. Proc Natl Acad Sci U S A. 2008;105:15100–5. 10.1073/pnas.0806301105 18812514 PMC2567499

[31] Rodrigues CDA, Ramírez-Guadiana FH, Meeske AJ, Wang X, Rudner DZ. GerM is required to assemble the basal platform of the SpoIIIA–SpoIIQ transenvelope complex during sporulation in Bacillus subtilis. Mol Microbiol. 2016;102:260–73. 10.1111/mmi.13457 27381174 PMC5055438

[32] Perez AR, Abanes-De Mello A, Pogliano K. SpoIIB localizes to active sites of septal biogenesis and spatially regulates septal thinning during engulfment in Bacillus subtilis. J Bacteriol. 2000;182:1096–108. 10.1128/jb.182.4.1096-1108.2000 10648537 PMC94387

[33] Aung S, Shum J, Abanes-De Mello A, Broder DH, Fredlund-Gutierrez J, Chiba S, et al. Dual localization pathways for the engulfment proteins during Bacillus subtilis sporulation. Mol Microbiol. 2007;65:1534–46. 10.1111/j.1365-2958.2007.05887.x 17824930 PMC2885130

[34] Salvado B, Vilaprinyo E, Sorribas A, Alves R. A survey of HK, HPt, and RR domains and their organization in two-component systems and phosphorelay proteins of organisms with fully sequenced genomes. PeerJ. 2015;3:e1183. Available from: https://peerj.com/articles/1183 26339559 10.7717/peerj.1183 PMC4558063

[35] De Hoon MJL, Eichenberger P, Vitkup D. Hierarchical evolution of the bacterial sporulation network. Curr Biol. Elsevier Ltd; 2010;20:R735–45. Available from: 10.1016/j.cub.2010.06.031 20833318 PMC2944226

[36] Martinez-Amador P, Castañeda N, Loza A, Soto L, Merino E, Maria R, et al. Prediction of protein architectures involved in the signaling—pathway initiating sporulation in Firmicutes. BMC Res Notes. BioMed Central; 2019;10–2. 10.1186/s13104-019-4712-3 31647017 PMC6813101

[37] Davidson P, Eutsey R, Redler B, Hiller NL, Laub MT, Durand D. Flexibility and constraint: Evolutionary remodeling of the sporulation initiation pathway in Firmicutes. PLoS Genet. 2018;14:1–33. 10.1371/journal.pgen.1007470 30212463 PMC6136694

[38] Burbulys D, Trach KA, Hoch JA. Initiation of sporulation in B. subtilis is controlled by a multicomponent phosphorelay. Cell. 1991;64:545–52. 10.1016/0092-8674(91)90238-t 1846779

[39] Kanehisa M. KEGG Organisms: Complete Genomes. KEGG as a Ref. Resour. gene protein Annot. 2015. p. d457–62. https://www.genome.jp/kegg/catalog/org_list.html

[40] Kanehisa M, Sato Y, Kawashima M, Furumichi M, Tanabe M. KEGG as a reference resource for gene and protein annotation. Nucleic Acids Res. 2016;44:D457–62. 10.1093/nar/gkv1070 26476454 PMC4702792

[41] Prakash A, Jeffryes M, Bateman A, Finn RD. The HMMER Web Server for Protein Sequence Similarity Search. Curr Protoc Bioinforma. 2017;60:3.15.1–3.15.23. 10.1002/cpbi.40 29220076

[42] El-Gebali S, Mistry J, Bateman A, Eddy SR, Luciani A, Potter SC, et al. The Pfam protein families database in 2019. Nucleic Acids Res. 2019;47:D427–32. 10.1093/nar/gky995 30357350 PMC6324024

[43] Levdikov VM, Blagova E V., McFeat A, Fogg MJ, Wilson KS, Wilkinson AJ. Structure of components of an intercellular channel complex in sporulating Bacillus subtilis. Proc Natl Acad Sci U S A. 2012;109:5441–5. 10.1073/pnas.1120087109 22431604 PMC3325715

[44] Meisner J, Moran CP. A LytM domain dictates the localization of proteins to the mother cell-forespore interface during bacterial endospore formation. J Bacteriol. 2011;193:591–8. 10.1128/JB.01270-10 21097616 PMC3021227

[45] Li W, Godzik A. Cd-hit: A fast program for clustering and comparing large sets of protein or nucleotide sequences. Bioinformatics. 2006;22:1658–9. 10.1093/bioinformatics/btl158 16731699

[46] Edgar RC. MUSCLE: Multiple sequence alignment with high accuracy and high throughput. Nucleic Acids Res. 2004;32:1792–7. 10.1093/nar/gkh340 15034147 PMC390337

[47] Cruz J, Liu Y, Liang Y, Zhou Y, Wilson M, Dennis JJ, et al. BacMap: An up-to-date electronic atlas of annotated bacterial genomes. Nucleic Acids Res. 2012 [cited 2019 Aug 23];40:599–604. Available from: https://academic.oup.com/nar/article-lookup/doi/10.1093/nar/gkr1105 22135301 10.1093/nar/gkr1105 PMC3245156

[48] Mukherjee S, Stamatis D, Bertsch J, Ovchinnikova G, Katta HY, Mojica A, et al. Genomes OnLine database (GOLD) v.7: Updates and new features. Nucleic Acids Res. 2019;47:D649–59. 10.1093/nar/gky977 30357420 PMC6323969

[49] Wattam AR, Davis JJ, Assaf R, Boisvert S, Brettin T, Bun C, et al. Improvements to PATRIC, the all-bacterial Bioinformatics Database and Analysis Resource Center. Nucleic Acids Res. 2017 [cited 2019 Aug 23];45:D535–42. Available from: https://academic.oup.com/nar/article-lookup/doi/10.1093/nar/gkw1017 27899627 10.1093/nar/gkw1017 PMC5210524

[50] Altschul SF, Gish W, Miller W, Myers EW, Lipman DJ. Basic local alignment search tool. J Mol Biol. 1990; 10.1016/S0022-2836(05)80360-2 2231712

[51] Gotelli NJ. Null Model Analysis of Species Co-Occurrence Patterns. Ecology. 2000;81:2606.

[52] Nguyen LT, Schmidt HA, Von Haeseler A, Minh BQ. IQ-TREE: A fast and effective stochastic algorithm for estimating maximum-likelihood phylogenies. Mol Biol Evol. 2015;32:268–74. 10.1093/molbev/msu300 25371430 PMC4271533

[53] Letunic I, Bork P. Interactive Tree Of Life (iTOL) v4: recent updates and new developments. Nucleic Acids Res. Oxford University Press; 2019;47:W256–9. 10.1093/nar/gkz239 30931475 PMC6602468

[54] Cohen O, Ashkenazy H, Belinky F, Huchon D, Pupko T. GLOOME: Gain loss mapping engine. Bioinformatics. 2010;26:2914–5. 10.1093/bioinformatics/btq549 20876605

[55] Trouve J, Mohamed A, Leisico F, Contreras-Martel C, Liu B, Mas C, et al. Structural characterization of the sporulation protein GerM from Bacillus subtilis. J Struct Biol. Elsevier; 2018;204:481–90. Available from: 10.1016/j.jsb.2018.09.010 30266596

[56] Notebaart RA, Huynen MA, Teusink B, Siezen RJ, Snel B. Correlation between sequence conservation and the genomic context after gene duplication. Nucleic Acids Res. 2005;33:6164–71. 10.1093/nar/gki913 16257980 PMC1275583

[57] Vos P, Garrity G, Jones D, Krieg NR, Ludwig W, Rainey FA, et al. Bergey’s manual of systematic bacteriology: Volume 3: The Firmicutes. Springer Science & Business Media; 2011.

[58] Oliver A, Kay M, Cooper KK. Comparative genomics of cocci-shaped Sporosarcina strains with diverse spatial isolation. BMC Genomics. BMC Genomics; 2018;19:1–17. 10.1186/s12864-017-4368-0 29716534 PMC5930826

[59] Kalmath BS, Prabhuraj A, Dhakephalkar PK, Hegde S. Characterization of Lysinibacillus sphaericus C3-41 strain isolated from northern Karnataka, India that is toxic to mosquito larvae. J Biol Control. 2014;28:24–30.

[60] Gao C, Han J, Liu Z, Xu X, Hang F, Wu Z. Paenibacillus bovis sp. nov., isolated from raw yak (Bos grunniens) milk. Int J Syst Evol Microbiol. 2016. 10.1099/ijsem.0.000900 26769366

[61] Edgar R. Taxonomy annotation and guide tree errors in 16S rRNA databases. PeerJ. 2018;2018. 10.7717/peerj.5030 29910992 PMC6003391

[62] Poehlein A, Yutin N, Daniel R, Galperin MY. Proposal for the reclassification of obligately purine-fermenting bacteria clostridium acidurici (Barker 1938) and clostridium purinilyticum (dürre et al. 1981) as gottschalkia acidurici gen. nov. comb. nov. and gottschalkia purinilytica comb. nov. and of. Int J Syst Evol Microbiol. 2017;67:2711–9. 10.1099/ijsem.0.002008 28853681 PMC5737214

[63] Yutin N, Galperin MY. A genomic update on clostridial phylogeny: Gram-negative spore formers and other misplaced clostridia. Environ Microbiol. 2013;15:2631–41. 10.1111/1462-2920.12173 23834245 PMC4056668

[64] Antunes LCS, Poppleton D, Klingl A, Criscuolo A, Dupuy B, Brochier-Armanet C, et al. Phylogenomic analysis supports the ancestral presence of LPS-outer membranes in the firmicutes. Elife. 2016;5:1–21. 10.7554/eLife.14589 27580370 PMC5007114

[65] Weller C, Wu M. A generation-time effect on the rate of molecular evolution in bacteria. Evolution (N Y). 2015;69:643–52. 10.1111/evo.12597 25564727

[66] Galperin MY, Brover V, Tolstoy I, Yutin N. Phylogenomic analysis of the family peptostreptococcaceae (Clostridium cluster xi) and proposal for reclassification of Clostridium litorale (Fendrich et al. 1991) and Eubacterium acidaminophilum (Zindel et al. 1989) as peptoclostridium litorale gen. nov. Int J Syst Evol Microbiol. 2016;66:5506–13. 10.1099/ijsem.0.001548 27902180 PMC5244501

[67] Stadtman TC, Mcclung LS. Clostridium sticklandii nov. spec. J Bacteriol. 1957;73:218–9. 10.1128/JB.73.2.218-219.1957 13416172 PMC289777

[68] Watanabe M, Kojima H, Fukui M. Complete genome sequence and cell structure of Limnochorda pilosa, a Gram-negative spore-former within the phylum Firmicutes. Int J Syst Evol Microbiol. 2016;66:1330–9. 10.1099/ijsem.0.000881 26743010

[69] Kunisawa T. Evolutionary relationships of completely sequenced Clostridia species and close relatives. Int J Syst Evol Microbiol. 2015;65:4276–83. 10.1099/ijsem.0.000638 26410691

[70] Rosero JA, Killer J, Sechovcová H, Mrázek J, Benada O, Fliegerová K, et al. Reclassification of Eubacterium rectale (Hauduroy et al. 1937) prévot 1938 in a new genus agathobacter gen. nov. as Agathobacter rectalis comb. nov., and description of Agathobacter ruminis sp. nov., isolated from the rumen contents of sheep and cows. Int J Syst Evol Microbiol. 2016;66:768–73. 10.1099/ijsem.0.000788 26619944

[71] Shetty SA, Zuffa S, Bui TPN, Aalvink S, Smidt H, De Vos WM. Reclassification of eubacterium hallii as Anaerobutyricum hallii gen. nov., comb. nov., and description of Anaerobutyricum soehngenii sp. nov., a butyrate and propionate-producing bacterium from infant faeces. Int J Syst Evol Microbiol. 2018;68:3741–6. 10.1099/ijsem.0.003041 30351260

[72] Taib N, Megrian D, Witwinowski J, Adam P, Poppleton D, Borrel G, et al. Genome-wide analysis of the Firmicutes illuminates the diderm/monoderm transition. Nat Ecol Evol. Springer US; 2020; 10.1038/s41559-020-01299-7 33077930

[73] Yang G, Chen J, Zhou S. Novibacillus thermophilus gen. Nov., sp. nov., a gram-staining-negative and moderately thermophilic member of the family Thermoactinomycetaceae. Int J Syst Evol Microbiol. 2015;65:2591–7. 10.1099/ijs.0.000306 25951858

[74] van Gestel J, Ackermann M, Wagner A. Microbial life cycles link global modularity in regulation to mosaic evolution. Nat Ecol Evol. Springer US; 2019;3:1184–96. Available from: 10.1038/s41559-019-0939-6 31332330

[75] Gupta RS, Patel S. Robust Demarcation of the Family Caryophanaceae (Planococcaceae) and Its Different Genera Including Three Novel Genera Based on Phylogenomics and Highly Specific Molecular Signatures. Front Microbiol. 2020;10. 10.3389/fmicb.2019.02821 PMC6971209 32010063

[76] Traag BA, Pugliese A, Eisen JA, Losick R. Gene conservation among endospore-forming bacteria reveals additional sporulation genes in Bacillus subtilis. J Bacteriol. 2013;195:253–60. 10.1128/JB.01778-12 23123912 PMC3553846

[77] Battistuzzi FU, Feijao A, Hedges SB. A genomic timescale of prokaryote evolution: Insights into the origin of methanogenesis, phototrophy, and the colonization of land. BMC Evol Biol. 2004;4:1–14. 15535883 10.1186/1471-2148-4-44 PMC533871

[78] Freyre-González JA, Manjarrez-Casas AM, Merino E, Martinez-Nuñez M, Perez-Rueda E, Gutiérrez-Ríos R-M. Lessons from the modular organization of the transcriptional regulatory network of Bacillus subtilis. BMC Syst Biol. 2013;7. 10.1186/1752-0509-7-127 24237659 PMC4225672

