# Supplementary figures and images for: Distribution and preservation of the components of the engulfment. What is beyond representative genomes?
Source: PLoS One. 2021 Mar 2;16(3):e0246651. doi: 10.1371/journal.pone.0246651 (PMC7924749; doi:10.1371/journal.pone.0246651)

# Unknown

## Class

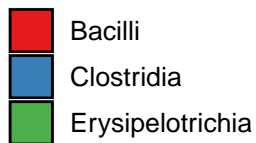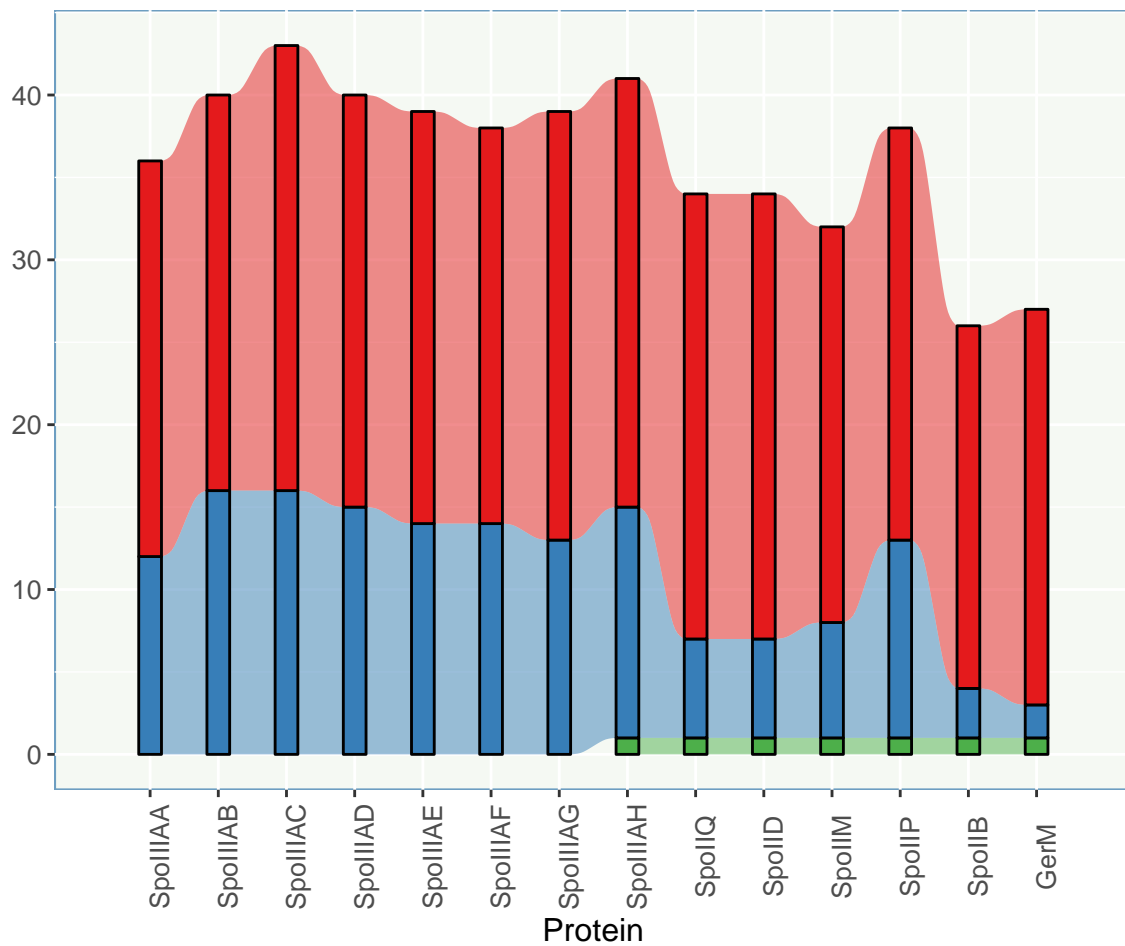

Supplement: S1 Fig — (PDF) [file pone.0246651.s002.pdf]
